# Metal‐Mediated Chlorine Transfer for Molten Salt‐Driven Thermodynamic Change on Silicon Production

**DOI:** 10.1002/advs.202412239

**Published:** 2024-12-03

**Authors:** Minjun Je, Jin Chul Kim, Jiyeon Kim, Sungho Kim, Sunmin Ryu, Jaegeon Ryu, Sang Kyu Kwak, Soojin Park

**Affiliations:** ^1^ Department of Chemistry Pohang University of Science and Technology (POSTECH) Pohang 37673 Republic of Korea; ^2^ School of Energy and Chemical Engineering Ulsan National Institute of Science and Technology (UNIST) Ulsan 44919 Republic of Korea; ^3^ Department of Chemical and Biomolecular Engineering Sogang University Seoul 04107 Republic of Korea; ^4^ Department of Chemical and Biological Engineering Korea University Seoul 02841 Republic of Korea

**Keywords:** lithium‐ion batteries, molten salt‐based thermochemical reduction, oxophilic metal, silicon production, thermodynamic change

## Abstract

The development of silicon (Si) material poses a great challenge with profound technological advancements for semiconductors, photo/photoelectric systems, solar cells, and secondary batteries. Typically, Si production involves the thermochemical reduction of silicon oxides, where chloride salt additives help properly revamp the reaction mechanism. Herein, we unravel the chemical principles of molten AlCl_3_ salt in metallothermic reduction. Above its melting temperature (*T*_m_ ≈ 192 °C), three AlCl_3_ molecules coordinate with each metal (M) atom (e.g., conventional Al and Mg, or even thermodynamically unfeasible Zn) to form metal‐AlCl_3_ complexes, M(AlCl_3_)_3_. In the molten AlCl_3_ salt media, all complexes directly lead to the universal formation of AlOCl byproduct and as‐reduced Si spheres through internal Cl^*^ transfer during the reduction reaction. Intriguingly, highly oxophilic metal (i.e., Mg) establishes additional energetic shortcuts in reaction pathways, where AlCl_3_ directly detaches an oxygen atom, accompanied by strong metal‐oxygen interactions and Cl^*^ transfer within the same complex. Moreover, the thermodynamic stability of the metal‐AlCl_3_ complex residue (MAl_2_Cl_8_) and the microstructure of post‐treated Si do change according to the metal choice, imparting disparate physicochemical properties for Si. This work offers insights into the scalable production of tailored Si materials for industrial applications, along with cost‐effective operations at 250 °C.

## Graphical abstract

Silicon (Si) production via metallothermic reduction is revolutionized through molten AlCl_3_ salt, enabling cost‐effective operations at 250 °C. Emerged metal‐AlCl_3_ complexes drive internal chlorine transfer for thermodynamic shifts, regardless of the metal's oxidation tendency. Oxophilic metals open additional pathways, lowering the activation energy. The metal choice significantly affects the final Si properties, leading to versatile applications.

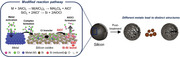

## 1 Introduction

Silicon (Si) element has sparked an incredible revolution in the green energy industry and information technology, serving as a cornerstone of human progress.^[^ ^1^, ^2^ ^]^ Physicochemical characteristics (e.g., optical, electronic, and mechanical properties) are influenced by the Si particle or feature size, which has usually been produced through the reduction of a naturally occurring form (i.e., silicon oxides) to meet market requirements.^[^ ^3^, ^4^, ^5^, ^6^ ^]^ Carbothermic reaction, which is one of the thermochemical reductions, is widely employed for oxygen elimination of SiO_2_ in the industry while requiring high temperatures over 1800 °C and yielding significant emission of pollutant gases (carbon monoxide, carbon dioxide, etc.).^[^ ^7^, ^8^ ^]^ When considering overall productivity and environmental impact, the metallothermic reduction method has emerged as an alternative approach to enable the removal of oxygen in SiO_2_ at a temperature above the melting point of metal reductants (*T*_m_ of Al ≈ 660 °C, Mg ≈ 650 °C, Zn ≈ 420 °C).^[^ ^9^, ^10^ ^]^ This method opens up large‐scale production of Si from a variety of precursors (earth‐abundant sources, industrial wastes, etc.) at a relatively low temperature compared to the traditional carbothermic reduction, offering cost‐effective production.^[^ ^11^ ^]^ However, highly accumulated exothermic heat during the thermochemical reduction accompanies the risk of explosion, and severely agglomerated products are likely to form.^[^ ^12^ ^]^ The adverse heat should be treated properly through various chemical and systematic approaches without disrupting the reaction.

For this purpose, chloride salt additives play a crucial role as heat scavengers in regulating the enormous exothermic heat throughout the system. The auxiliary salts (e.g., NaCl, KCl) absorb the generated heat during the reduction reaction and become molten, thus managing the overall kinetics but not participating in the reaction.^[^ ^13^, ^14^ ^]^ On the other hand, binary or ternary molten salts can establish a eutectic system with much lower melting points than individual components, which renders liquid media to accelerate the reaction with a decrease in the reduction temperature.^[^ ^15^ ^]^ Interestingly, specific single molten salt can make a reactive medium and directly engage in the reduction reaction. Qian group reported that the addition of aluminum chloride (AlCl_3_) salt in the metallothermic reduction expedited the reaction at a relatively low temperature of ≈250 °C.^[^ ^16^ ^]^ With an aluminum (Al) metal reductant, the melted AlCl_3_ (*T*_m_ of AlCl_3_ ≈ 192 °C) enables Al metal to dissolve in the molten salt matrix far below its melting temperature.^[^ ^17^ ^]^ The Al metal‐dissolved molten AlCl_3_ salt environment experienced internal rearrangement to produce AlCl^*^. This radical induced oxygen disconnection on the SiO_2_ surface, forming a new Si─Si bond and the primary byproduct of aluminum oxychloride (AlOCl). In addition, powerful AlCl^*^ reductant can reduce oxygen elements in other oxides, affording heteroatom‐infused and multifarious bonds‐impregnated Si from industrial wastes and low‐cost Si precursors.^[^ ^18^, ^19^ ^]^ Therefore, the reaction mechanism in the presence of AlCl_3_ salt not only expands the fundamental understanding of chemical processes but also offers practical implications for the advancement of sustainable and cost‐effective production methods.

**Figure 1:**
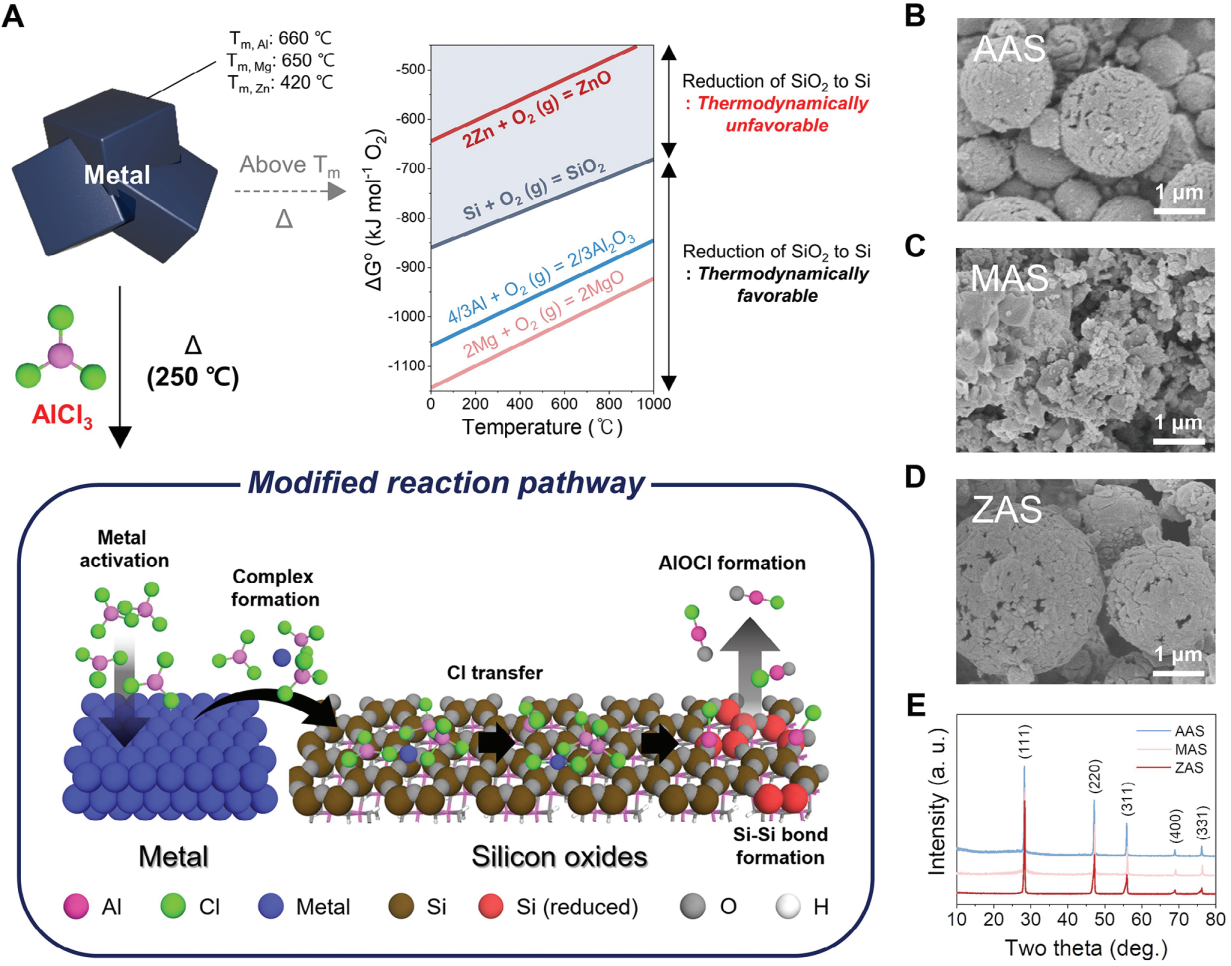
Unique chemistry of molten AlCl_3_ medium. A) Schematic illustration of AlCl_3_ molten salt‐modified metallothermic reduction reaction through universal Cl transfer. SEM images of B) AAS, C) MAS, and D) ZAS after post‐treatment. E) XRD patterns of AAS, MAS, and ZAS after post‐treatment. Atoms of surface and adsorbate are colored differently for a clear view. For the adsorbate, Al and Cl atoms in the ligand are colored in pink and light green, respectively. The metal and Si atoms are colored in blue and brown, and clustered Si, O, and H atoms are colored in scarlet, gray, and white, respectively.

AlCl_3_ molten salt‐modified reduction system has been extensively explored with different metal reductants, for example, activated magnesium (Mg) in AlCl_3_ molten salt can reduce SiCl_4_ and SiO_2_ to Si at low temperatures of 200 °C.^[^ ^20^, ^21^ ^]^ Mg metal accepted chlorine (Cl) atoms from AlCl_3,_ and thus metallic Al and MgCl_2_ were yielded through this Cl^*^ transfer, leading to the reduction reaction of SiCl_4_ occurring by nascent Al metal. The salt‐assisted magnesiothermic reduction of SiO_2_ affords a disparate byproduct (MgAl_2_Cl_8_) but no exact reaction pathway remains completely elucidated for this reduction system.^[^ ^22^ ^]^ For zinc (Zn) metal reductants, according to the Ellingham diagram (**Figure** **1**A), zincothermic reduction of SiO_2_ poses a thermodynamic challenge due to the non‐favorable oxidation tendency of metallic Zn compared to Si, thus limiting the available precursor (i.e., SiCl_4_) and requiring high energy (>1000 °C).^[^ ^23^ ^]^ Recently, Yin's group suggested that AlCl_3_ can trigger chlorination of SiO_2_ in the molten salt medium, in which a gaseous SiCl_4_ was reduced to Si by subsequent reaction with the metallic Zn.^[^ ^24^, ^25^ ^]^ This proposed Cl^*^ transfer enables a thermodynamically unfeasible reduction of SiO_2_ to be possible by driving the change in the molecular energy level, which modifies the reaction pathway. However, previous studies put forward inconsistent mechanisms involving individual metals for salt‐mediated reduction, making it challenging to isolate and fully understand the molten salt chemistry of AlCl_3_. In this regard, it is imperative to uncover the intrinsic function of AlCl_3_ salt in the molten salt‐modified reduction system while considering the universal byproduct of AlOCl in the solution regardless of metal reductants.

Herein, we propose the inherent roles of AlCl_3_ salt in the AlCl_3_ molten salt‐modified metallothermic reduction reaction of SiO_2_ utilizing common metal reductants. Through comprehensive experimental analysis and density functional theory (DFT) calculations, our investigation into the unique chemistry of AlCl_3_ salt unveils its capability to form metal (M)‐AlCl_3_ complexes, M(AlCl_3_)_3_. Notably, in the absence of AlCl_3_ salt, specific metals like Zn render the reduction of silicon oxides thermodynamically unfavorable. Nonetheless, AlCl_3_ salt formulates the metal‐AlCl_3_ complex, even with Zn metal, thereby facilitating thermodynamic changes for the reduction reaction to proceed. Within all complexes, an effective reducing agent (i.e., AlCl^*^) is generated through internal Cl^*^ transfer, redefining the role of all metals from an oxygen detacher to a Cl acceptor. This radical then disengages one oxygen atom from SiO_2_, yielding the uniform byproduct of AlOCl with self‐assembled Si spheres. Meanwhile, reactive metals to oxygen (i.e., Mg) could help AlCl_3_ molecules pioneer another reaction route for oxygen dissociation, based on metal‐oxygen interactions and identical Cl^*^ transfers.

Importantly, the final Si morphology and the thermodynamic stability of the remnant for the metal‐AlCl_3_ complex (MAl_2_Cl_8_) vary depending on the metal deployed, resulting in structurally distinct Si, as evaluated by electrochemical analysis in lithium‐ion battery systems. Therefore, this study not only provides a deep understanding of molten AlCl_3_ salt chemistry but also establishes a practical pathway of the molten salt‐modified metallothermic reduction for scalable and high‐quality production of Si in numerous cutting‐edge industries.

## 2 Results and Discussion

### 2.1 AlCl_3_: A Game‐Changer in Reduction Reactions via Cl Transfer

The overall procedure of the Si‐Si bond conversion from the Si‐O‐Si layer through the thermochemical reduction reaction is illustrated in Figure 1A. First, the reduction mechanism on the silicon oxide surface was investigated by the DFT calculation provided that each metal would undergo solvation in molten salts to establish a complex below the melting points of metallic Mg (≈660 °C) and Zn (≈420 °C). The AlCl_3_ molten salt‐modified metallothermic reduction reaction occurs in two distinct stages; (i) the formation of a metal‐centered complex with AlCl_3_ salts, and (ii) the reduction of the silicon oxide promoted by the adhered complex. After adsorbing the metal‐AlCl_3_ complex (M(AlCl_3_)_3_) on the silicon oxide surface, the mechanism involves reduction not by the metal center, but rather by the activated AlCl^*^ generated through the Cl^*^ transfer from the ligand of the complex to the metal center. In creating the activated AlCl^*^, the AlCl_3_ ligand is solely separated from the metal‐AlCl_3_ complex, followed by successive two‐time Cl^*^ transfers. Segregating oxygen atoms by AlCl^*^ from the silicon oxide layer offers AlOCl as the byproduct and a new Si‐Si bond, altering the reaction's thermodynamics irrespective of the metal reductants exploited (Figures S1–S3, Supporting Information). Afterward, as‐generated many AlOCl molecules could provide a clustering environment for Si atoms to be Si seeds.

The newly formed Si seeds participate in a recrystallization process, where the growing intermediates should minimize surface energy to achieve thermodynamic stability. Consequently, the as‐reduced Si assembles into a spherical shape, which presents the lowest surface energy. Additionally, during the maturation phase, Ostwald ripening occurs as a thermodynamically‐driven spontaneous process. The Laplace pressure inside smaller droplets is higher than inside larger droplets, causing smaller, less stable Si particles to dissolve and re‐precipitate onto larger, energetically favored ones.^[^ ^26^ ^]^ Ultimately, Ostwald ripening leads to the formation of a stable outer shell, switching the solid structure into a hollow one.^[^ ^27^ ^]^ Note that the porous structure emerged following the reduction reaction and the clearance of byproducts. In this chemical environment, Al and Zn metals tend not to react preferentially with the nascent Si atom thanks to their inherent alloying properties. Therefore, following the leaching process, both aluminum/aluminum chloride‐reduced Si (AAS) and zinc/aluminum chloride‐reduced Si (ZAS) preserve their overall morphological frames, featuring hollow and porous Si spheres (Figure 1B–D). In contrast, dissolved Mg atoms exhibit rapid diffusion through the medium and thus readily react with the highly unstable Si seeds to formulate magnesium silicide.^[^ ^28^, ^29^ ^]^ With undesired but significant magnesiation of Si, the prevailing and strong coarsening of Si crumbs, coupled with non‐negligible magnesium silicide, also usher a spherical growth of magnesium/aluminum chloride‐reduced Si (MAS) similar to that observed in AAS and ZAS. These compositional differences would make structural variations in subsequent treatments.^[^ ^30^, ^31^ ^]^ Despite the varying reactivity of all the metals with Si, the structural maturation of Si seeds led to a crystalline organization, as validated by X‐ray diffraction (XRD) analysis of acid‐leached samples (Figure 1E).

According to a recent study proposed by Yin's group, the corresponding reduction reaction suggests that SiO_2_ undergoes a chlorination process to form SiCl_4_, rather than involving Cl^*^ transfer in a metal‐AlCl₃ complex, highlighting the different roles of Cl.^[^ ^24^, ^25^ ^]^ Importantly, in the absence of any metal sources, the reaction between SiO_2_ and AlCl_3_ salt does afford no discernible peaks apart from the crystalline peaks of AlCl_3_, with notably no AlOCl peaks detected in the XRD patterns (Figure S4A, Supporting Information). Moreover, we theoretically examined the above reaction using only molten Al_2_Cl_6_ molecules (Figure S4B,C, Supporting Information). Note that the initial step of the reduction reaction is characterized by the cleavage of the Al─Cl bond in the Al_2_Cl_6_ molecule, resulting in the attachment of Al_2_Cl_5_ and Cl to the SiO_2_ surface. This reaction requires a high activation energy (*E_a_*) of 4.31 eV and a heat of reaction (Δ*E*) of 2.69 eV, indicating that the SiO_2_ activation reaction by molten salts is kinetically and thermodynamically unfavorable. Furthermore, the process of fabricating the metal‐AlCl_3_ complex was theoretically studied with probable configurations of adsorbed salts and salt‐solvated complexes on metal surfaces (Figure S5, Supporting Information). Similar to the Al dissolution mechanism described in a previous study, ^[^ ^17^ ^]^ the *E_a_* and Δ*E* required for the Mg and Zn metal dissolution by AlCl_3_ molecules were compared. The Mg dissolution reaction displays an *E_a_* of 0.62 eV and Δ*E* of −0.28 eV, and the *E_a_* and Δ*E* for the Zn dissolution reaction are identified to be 0.94 and 0.63 eV, respectively. From these results, in molten salt‐incorporated reactions even with Mg and Zn metals, three AlCl_3_ molecules entirely interact with and thus dissolve the metal atom to form a metal‐AlCl_3_ complex. In addition, the construction of the metal‐AlCl_3_ complex and the succeeding Cl^*^ transfer are energetically more favorable compared to a simple chlorination reaction producing SiCl_4_ without metals.

### 2.2 Molten Salt‐Driven Thermodynamic Change with Strong Oxophilic Metal

**Figure 2:**
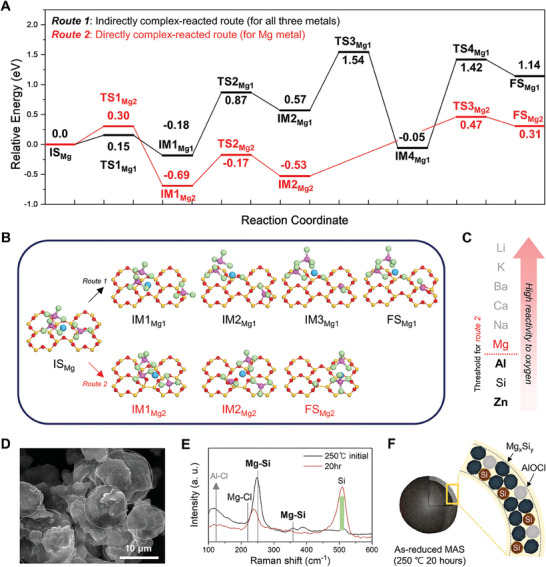
Molten salt‐driven thermodynamic change in the magnesiothermic reduction reaction. A) Reaction coordinate and B) IS_Mg_, IM_Mg_, and FS_Mg_ configurations of *routes 1* and *2* within AlCl_3_ molten salt‐modified magnesiothermic reduction. For a clear view, all atoms except the first layer of silicon oxide are omitted. For the color scheme of atoms, Mg, Al, and Cl atoms are colored in sky blue, pink, and light green, and Si and O atoms in the silicon oxide are colored in yellow, and red, respectively. C) Basic characteristics of metals for oxygen reactivity and threshold for *route 2*. D) SEM image of as‐reduced MAS particles. The as‐reduced MAS is identical to the sample of 250 °C 20 h without any post‐treatment. E) Raman spectra of samples participated in AlCl_3_ molten salt‐modified magnesiothermic reduction reaction for 250 °C initial, 20 h. F) Scheme for the chemical composition of the as‐reduced MAS following growth through Ostwald ripening and coalescence with Mg*_x_* Si*_y_*.

In the reduction reaction occurring on the silicon oxide surface, we traced the step‐by‐step mechanism facilitated by the metal‐AlCl_3_ complex, leading to the establishment of the Si‐Si bond and the AlOCl byproduct. First, the molten AlCl_3_ salt‐modified magnesiothermic reduction mechanism was investigated employing the Mg‐AlCl_3_ complex (**Figure** **2**A,B; Figure S6, Supporting Information). After the adsorption step of the Mg‐AlCl_3_ complex on the silicon oxide surface, the reduction reaction diverges into two pathways: an *indirectly complex‐reacted route*, where AlOCl is formed by segregating the AlCl_3_ ligand intermediate from the complex, and a *directly complex‐reacted route*, where the Mg atom and AlCl_3_ molecule in the complex directly detach the surface oxygen.

In the *indirectly complex‐reacted route*, the first step confirms that one AlCl_3_ ligand isolates itself from the Mg‐AlCl_3_ complex and solely adheres to the SiO_2_ surface. This ligand dissociation step has an *E_a_* of 0.15 eV and Δ*E* of −0.18 eV. Subsequently, in IM2_Mg1_, a Cl^*^ atom of the separated AlCl_3_ ligand moves into the Mg(AlCl_3_)_2_ complex, and the remaining AlCl_2_^*^ interacts with three oxygens on the silicon oxide surface, with an *E_a_* of 1.05 eV and Δ*E* of 0.75 eV. For the next step, the second Cl^*^ atom of AlCl_2_^*^ is further transferred to the complex, and the resultant AlCl^*^ attaches to the center of the 6‐membered ring on the silicon oxide surface, with an *E_a_* of 0.94 eV and Δ*E* of −0.62 eV. From IM3_Mg1_ to FS_Mg1_, as AlCl^*^ reacts with oxygen in the SiO_2_ surface, AlOCl is created through the cleavage of strong Si─O bonds with the highest *E_a_* of 1.47 eV and Δ*E* of 1.19 eV. In addition, the reduction reaction by the central Mg atom in the complex is examined as the *directly complex‐reacted route*. After adhering the complex to the silicon oxide surface, an oxygen atom in the silicon oxide surface could be desorbed by the AlCl_3_ ligand and Mg atom, rendering an Mg─O─AlCl_3_ bond with an *E_a_* of 0.30 eV and Δ*E* of −0.69 eV. From IM1_Mg2_ to IM2_Mg2_, the Cl^*^ atom, connected to the outermost oxygen, smoothly migrates to an adjacent AlCl_3_ ligand, with an *E_a_* of 0.52 eV and Δ*E* of 0.16 eV. Afterward, the secondary Cl^*^ atom from the AlCl_2_O also moves to the other AlCl_3_ ligand, and the Mg‐O interaction is broken with the highest *E_a_* of 1.00 eV and Δ*E* of 0.84 eV, yielding a universal AlOCl byproduct and the same complex. Based on the activation energy at the rate‐determining step for both the indirect and direct reaction pathways, all the routes are feasible for the actual reaction under molten salt conditions. Importantly, the Mg‐activated reduction mechanism (i.e., *route2: directly complex‐reacted route*) offers an additional, kinetically favorable pathway, whereas all metals, including Mg, participate in *route 1*. The supplementary *route 2* becomes a viable reaction pathway when the interaction between the metal and oxygen is sufficiently strong (Figure 2C).^[^ ^32^, ^33^ ^]^

Interestingly, after the above reduction reaction and recrystallization process, the post‐treated MAS possesses a nanoparticulate structure as shown in Figure 1C. However, as‐reduced MAS also grows into micrometer spheres ranging from a few µm to 10 µm with a rough surface through Ostwald ripening, analogous to AAS and ZAS (Figure 2D; Figure S7, Supporting Information). Notably, upon reaching 250 °C, the Raman spectra prove that the peaks for Mg‐Si are more prevalent at 251 and 358 cm^−1^ than those for Si─Si at 500–520 cm^−1^ (Figure 2E).^[^ ^34^ ^]^ In addition to the anterior peak bump of Al─Cl bonds between 100 and 200 cm⁻¹, and the inconspicuous peaks ≈300 cm⁻¹, Mg metal displays relatively higher chemical reactivity than Al and Zn metals, enabling it to easily formulate Mg─Si bonds at temperatures as low as room temperature and the operating temperature of 250 °C. (Figure S8, Supporting Information).^[^ ^35^, ^36^ ^]^ Over 20 h, the Mg‐Cl peak becomes prominent at 217 cm^−1^ along with the peak for the Si─Si bond due to extensive reaction progress and the formation of the final complex state, MgAl_2_Cl_8_.^[^ ^37^ ^]^ Above the melting point of AlCl_3_, a considerable portion of nascent Si atoms occurs in side reactions with Mg to create magnesium silicide when Si atoms cluster into Si seeds. Concurrently, small Si crystal seeds, magnesium silicide, and generated AlOCl are embedded together with remaining AlCl_3_ salts in a Si sphere through self‐assembly driven by Ostwald ripening (Figure 2F).^[^ ^31^ ^]^ The co‐amalgamation of substantial Mg─Si bonds in the as‐produced MAS prompts vigorous reactions with acids like HCl and melting down during the leaching step, causing the physical disintegration of the sphere and thus converting the Si crumbs that constituted the sphere into MAS.

### 2.3 Molten Salt‐Driven Thermodynamic Change with Weak Oxophilic Metal

**Figure 3:**
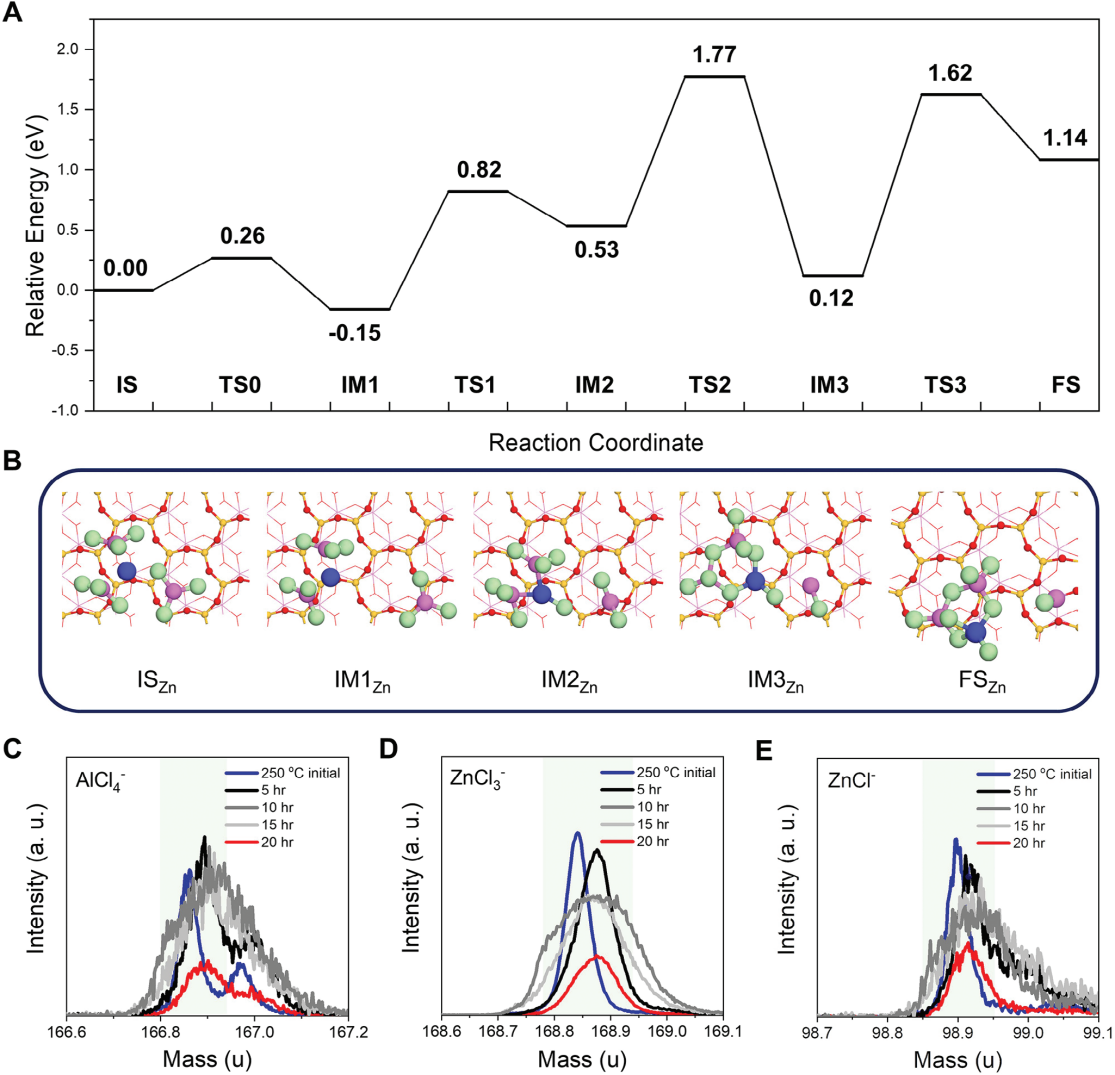
Molten salt‐driven thermodynamic change in the zincothermic reduction reaction. A) Theoretically tracked reaction mechanism and B) IS_Zn_, IM_Zn_, and FS_Zn_ configurations of AlCl_3_ molten salt‐modified zincothermic reduction. For the color scheme of atoms, Zn, Al, and Cl atoms are colored in blue, pink, and light green, and Si, O, and H atoms in the silicon oxide are colored in yellow, red, and white, respectively. The numbers represent the relative energies of each state based on that of the IS. TOF‐SIMS analysis to C) AlCl_4_^−^, D) ZnCl_3_^−^, and E) ZnCl^−^ of the samples participated in the reduction reaction for 250 °C initial, 5, 10, 15, and 20 h.

Without AlCl_3_ salt, less oxidative metals such as Zn cannot proceed with the reduction of silicon oxides. However, within a molten AlCl_3_ matrix, the as‐generated metal‐AlCl₃ complexes induce consecutive thermodynamic changes regardless of the metals deployed, as shown in Figure 1A. Therefore, the molten salt‐modified zincothermic reduction mechanism is also validated to have two pathways through DFT calculations: the *indirectly complex‐reacted route* and the *direct complex‐reacted route* (**Figure** **3**A,B; Figure S9, Supporting Information). However, the directly complex‐reacted reduction mechanism by the Zn‐AlCl_3_ complex is not observed. It is demonstrated that the surface oxygen atom returns to the silicon oxide surface when the oxygen atom is about to detach, resulting from differences in interactions between metal‐oxygen and metal‐ligand bonds. The other pathway involves the same Cl^*^ transfer, which continues to create AlOCl. In the first intermediate state, with an *E_a_* of 0.26 eV and Δ*E* of −0.15 eV, the AlCl_3_ ligand is solely separated from the [Zn(AlCl_3_)_3_] complex and adsorbs on the SiO_2_ surface. Subsequently, in IM2 _Zn_, a Cl^*^ atom is transferred from the disengaged AlCl_3_ ligand to the Zn‐AlCl_3_ complex ([Zn(AlCl_3_)_2_]), and the remaining AlCl_2_^*^ interacts with two oxygens on the silicon oxide surface with an *E_a_* of 0.97 eV and Δ*E* of 0.68 eV. From the IM2 _Zn_ step to the IM3 _Zn_ step, a Cl^*^ atom moves once more from AlCl_2_^*^, similarly to the previous reaction step, forming an AlCl^*^ intermediate with an *E_a_* of 1.24 eV and Δ*E* of –0.41 eV. In the next step, the remaining AlCl^*^ intermediate attached to the surface facilitates the segregation of one surface oxygen atom to form the AlOCl byproduct with the highest *E_a_* (1.50 eV) and Δ*E* (1.02 eV), thereby serving as the rate‐determining step in the molten salt‐mediated reduction process at 250 °C.

Concurrently, time‐of‐flight secondary ion mass spectrometry (TOF‐SIMS) analysis confirmed the presence of molecular bonding including multiple atoms of Al, Zn, and Cl. Experimental evidence reveals direct covalent bonds between Zn and AlCl_3_ by initiating from IM2 _Zn_, predominantly featuring AlCl_4_^−^ and ZnCl_3_^−^ in the Zn‐AlCl_3_ complex (Figure 3C,D). Even upon reaching 250 °C, strong signals of AlCl_4_^−^ and ZnCl_3_^−^ are detected, indicating an early and easy construction of the Zn‐AlCl_3_ complex. Afterward, the molecular binding of AlCl_4_^−^ and ZnCl_3_^−^ shows sustained prominence in the spectra for up to 15 h. With the termination of the reduction reaction after 20 h, the further establishment of the Zn‐AlCl_3_ complex is no longer necessary. Importantly, the binding peaks of AlCl_4_^−^ and ZnCl_3_^−^, as well as ZnCl^−^ within the complex also displayed a marked decrease (Figure 3E). While the original form of the two Cl atoms‐accepted Zn‐AlCl_3_ complex corresponds to ZnAl_2_Cl_8_, the end‐stage complex fails to maintain this chemical structure, implying a subsequent internal rearrangement.

### 2.4 Internal Division of Metal‐AlCl_3_ Complex Due to Thermodynamic Instability

**Figure 4:**
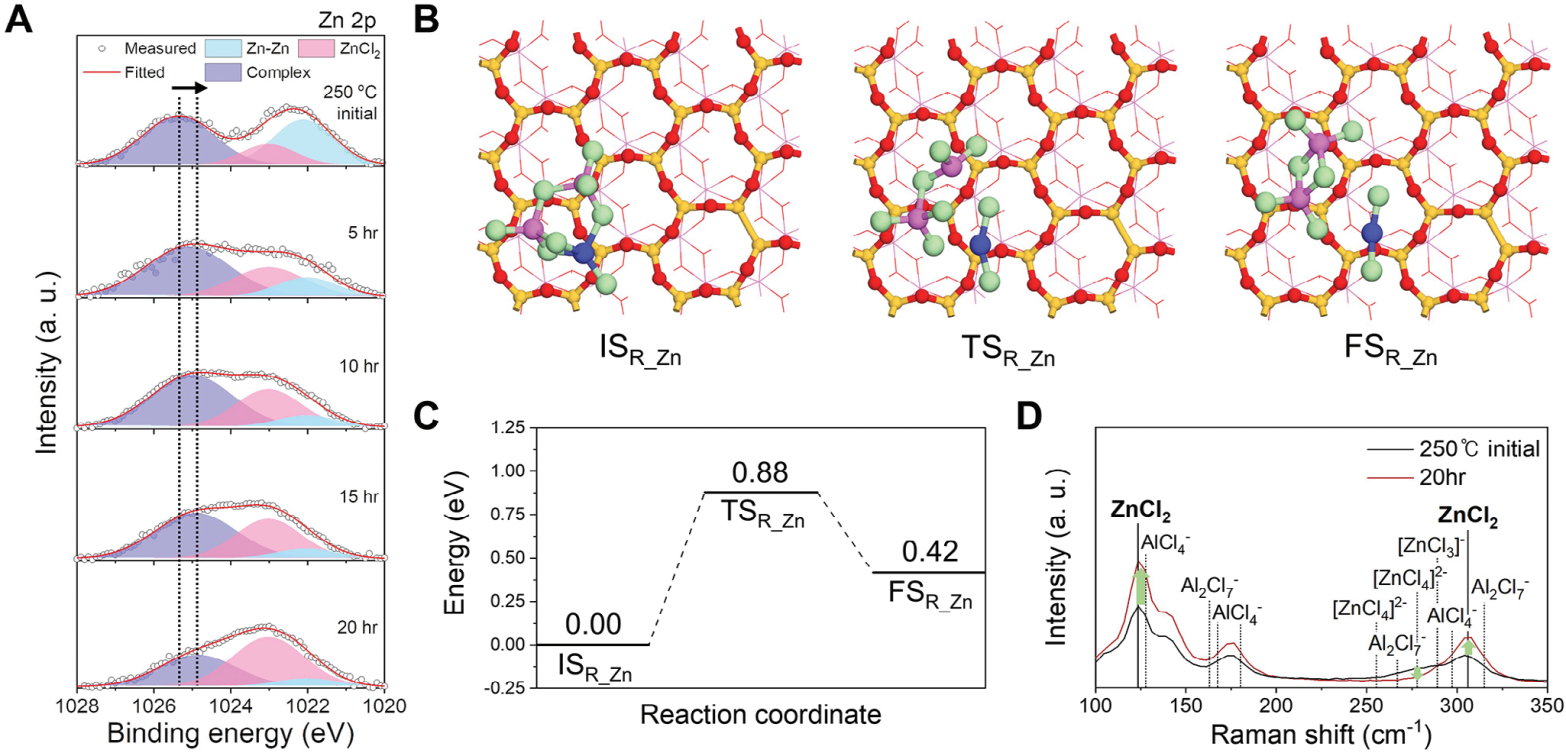
Self‐rearrangement in Zn‐AlCl_3_ complex. A) Zn 2p XPS spectra of samples participated in AlCl_3_ molten salt‐modified zincothermic reduction reaction for 250 °C initial, 5, 10, 15, and 20 h. B) Configurations and C) reaction coordinate for following structural rearrangement of two Cl atoms‐accepted Zn‐AlCl_3_ complex to produce ZnCl_2_. The color scheme of atoms is same as in Figure 3. D) Raman spectra of samples participated in AlCl_3_ molten salt‐modified zincothermic reduction reaction for 250 °C initial, 20 h.

For an in‐depth investigation of the reaction‐undergone Zn‐AlCl_3_ complex and its following structural changes, X‐ray photoelectron spectroscopy (XPS) measurements were conducted on as‐reduced samples that were controlled for reaction time (**Figure** **4**A; Figure S10, Supporting Information). At the early stage, the Zn‐AlCl_3_ complex is primarily in a state where it is bound with an abundance of Cl, resulting in a higher binding energy.^[^ ^38^, ^39^ ^]^ Through the additional reaction, the final product, [ZnAl_2_Cl_8_], in the reduction, is converted to ZnCl_2_ and Al_2_Cl_6_ due to the Cl^*^ transfer with the *E_a_* of 0.88 eV and Δ*E* of 0.42 eV (Figure 4B,C). Therefore, even at the temperature reaching 250 °C, the presence of ZnCl_2_ at 1023 eV in Zn 2p XPS spectra suggests that structural reformation is facile, which indicates a preferred rearrangement toward the production of ZnCl_2_.^[^ ^40^ ^]^ Interestingly, after the molten salt‐modified magnesiothermic reduction, the rearrangement reaction does not occur from the complex products to MgCl_2_. To theoretically confirm this observation, the formation energies of each metal halide and metal‐AlCl_3_ complex are calculated (Figure S11, Supporting Information). In this result, the formation energy of ZnCl_2_ + 2AlCl_3_ is higher than that of the product, [ZnAl_2_Cl_8_], so the rearrangement reaction seems feasible. However, given the higher structural stability of the MgAl_2_Cl_8_ compared to MgCl_2_ + 2AlCl_3_, no rearrangement of the MgAl_2_Cl_8_ complex is observed, which is consistent with the absence of MgCl_2_ peaks in the XRD pattern, and the non‐dominant Mg─Cl peaks in the Raman spectra.

Additionally, the time‐controlled XRD patterns unveil the dissolution of crystalline Zn and AlCl_3_ at 43.5 and 15.2–15.3° which implies their engagement in the reaction, while peaks associated with AlOCl, ZnCl_2_, and Si are gradually developed at 10.7–10.9, 28.3, and 28.4°, respectively (Figure S3, Supporting Information).^[^ ^16^, ^41^ ^]^ Eventually, the end‐stage Zn‐AlCl_3_ complex in the form of ZnAl_2_Cl_8_ dissociates into ZnCl_2_, consistent with a shift toward lower binding energy in Zn 2p and Al 2p XPS spectra (Figure 4A; Figure S10, Supporting Information). The samples are denoted as INT‐1 for the as‐reduced sample, INT‐2 for the water‐treated sample, and the final sample for the HCl‐treated sample. Upon immediately reaching 250 °C, the XRD pattern of water‐treated (INT‐2) samples provides critical evidence for the production of ZnCl_2_ (Figure S12, Supporting Information).^[^ ^42^, ^43^ ^]^ Residual nanosized Zn metal that remained unreacted during the reduction process undergoes partial oxidation to ZnO upon exposure to air or moisture, as considerable heat is generated by exothermic reactions during AlCl_3_‐dissolved water treatment. The subsequent chain reactions can be represented as follows:^[^ ^44^ ^]^

(1) $$\mathrm{ZnC}l_{2}+\mathrm{ZnO}+H_{2}O\to 2\beta -\mathrm{Zn}\left(\mathrm{OH}\right)\mathrm{Cl}$$

(2) $$\begin{array}{ccc} & & 2\beta -\mathrm{Zn}\left(\mathrm{OH}\right)\mathrm{Cl}+3\mathrm{ZnO}+4H_{2}O\to 2/5Zn_{5}{\left(\mathrm{OH}\right)}_{8}Cl_{2}\cdot \left(H_{2}O\right)\\ & & +6/5\mathrm{HCl}+8/5H_{2}O \end{array}$$

After a certain reaction time of less than 5 h, the small remaining quantity of Zn metal can be completely removed in the acidic environment created by the dissolution of AlCl_3_ in INT‐2. Consequently, little ZnO is formed at 32.0 and 34.6° with large exothermic heat, yielding undetectable levels of Zn metal. Raman analysis is a non‐destructive strategy for not only intramolecular ionic bonds but also a high‐mass species of Al_2_Cl_7_^−^, while the TOF‐SIMS technique makes it difficult to verify an oxidation state of the detected species and internal bonds with multiply charged ions such as ZnCl_4_^2−^.^[^ ^45^, ^46^ ^]^ Without any potential for ionization of the complex, the advanced approach proves the internal rearrangement in the complex (Figure 4D). AlCl_4_^−^ is present at 127, 167, 180, and 297 cm^−1^ in the form of ZnAl_2_Cl_8_ and Al_2_Cl_6_ before and after rearrangement, and its absolute amount increases over reaction time, respectively.^[^ ^47^ ^]^ However, components related to the Zn‐AlCl_3_ complex such as Al_2_Cl_7_^−^ at 163, 267, and 315 cm^−1^, ZnCl_3_^−^ at 289 cm^−1^, and ZnCl_4_^2−^ at 255 and 278 cm^−1^ almost disappear whereas ZnCl_2_ significantly emerges at 123 and 306 cm^−1^, which is consistent with the theoretical calculation results.^[^ ^48^ ^]^ These transformative phenomena would give rise to the manifestation of structural differences.

### 2.5 Structural Difference by Metal Selection

**Figure 5:**
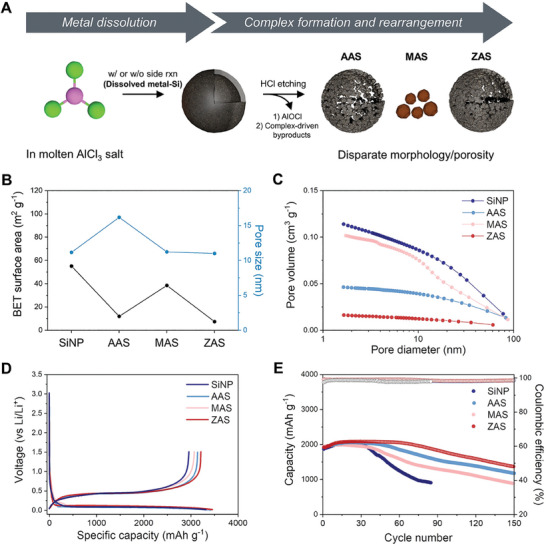
Comparative analysis for distinctive structural properties of AAS, MAS, and ZAS with an application in lithium‐ion battery system. A) Schematic illustration of factors influencing Si coalescence and resulting Si structure derived from AlCl_3_ molten salt‐modified metallothermic reduction reaction. B) BET surface area values, pore size, and C) pore volumes of SiNP, AAS, MAS, and ZAS samples. D) Galvanostatic charge/discharge profiles and E) cycling stability of the electrodes at 0.5C. All structural analyses were conducted on HCl‐treated AAS, MAS, and ZAS.

The introduction of AlCl_3_ into metallothermic reduction reactions fundamentally changes the reaction mechanism, pathways, and chemical principles. This universal chemistry of AlCl_3_ holds across all metal sources, consistently leading to the adsorption and combination of molten AlCl_3_ molecules on the metal surface, and activated metal‐mediated Cl^*^ transfer. Importantly, each employed metal influences the number of reaction pathways, the thermodynamic stability of the complex residue, and the structural characteristics of synthesized Si (**Figure** **5**A). Both with and without side reactions between the metal and nascent Si seeds, the spherical Si structures emerge in all the AAS, MAS, and ZAS particles. The post‐treated AAS and ZAS exhibit different sphere porosities due to variations in byproducts after self‐rearrangement of the complex. However, in as‐reduced MAS, a significant portion comprises magnesium silicide, which dissolves during post‐treatment, leading to structural collapse and the creation of pure Si nanoparticles as the remaining building blocks (Figure S13, Supporting Information).

In the molten AlCl_3_ salt environment, the morphology of the grown Si is significantly influenced by the heat treatment conditions. SEM analysis, conducted at different duration times at 250 °C, illustrates a diversity of ZAS shapes and sizes with broken spheres after post‐treatment (Figures S14 and S15, Supporting Information). The Si seeds lack sufficient thermal energy to establish hollow spheres and the transformation of surface atoms in a finite crystal.^[^ ^49^ ^]^ A reaction duration of 20 h proved optimal for the growth of all Si seeds into similarly sized spheres. Notably, immediately upon reaching 250 °C and cooling down, analysis detected a significant 5.78% of Zn for water‐dissolved ZAS in INT‐2 (Figure S16, Supporting Information), consistent with the XRD pattern of the same sample in Figure S12 (Supporting Information). However, Zn metal is no longer discernible from a duration of 5 h onward, and only water‐treated ZAS achieves over 90% purity of Si with a reaction time extended to 20 h in INT‐2. Furthermore, time‐resolved XRD analysis of the HCl‐treated final samples reveals a remarkable increase in crystallite size at 20 h of duration (Figure S17, Supporting Information). A multifarious analysis of SEM, elemental characterization, and XRD collectively demonstrate the completion of both the Si growth process and reduction reaction.

Based on the above morphological features, the precise structural evolution of each reduced final material was investigated through Brunauer−Emmett−Teller (BET) and Barrett‐Joyner‐Halenda (BJH) analysis. Compared to commercial 50 nm SiNP, the relatively larger‐sized MAS represented in the hundreds of nanometers, and thus the BET surface area of MAS tends to be smaller than SiNP, providing 55.117 m^2^ g^−1^ for SiNP and 38.499 m^2^ g^−1^ for MAS, respectively (Figure 5B; Figure S18, Supporting Information). Likewise, AAS and ZAS show differences in pore sizes, surface areas, and pore volumes due to dissimilar metal centers and corresponding rearrangements. AAS displayed a BET surface area of 11.921 m^2^ g^−1^ and ZAS of 7.406 m^2^ g^−1^, with pore sizes of 16.172 and 10.998 nm, respectively. ZAS provides much denser pores with a smaller pore volume of 0.0158 cm^3^ g^−1^ compared to 0.0463 cm^3^ g^−1^ for AAS (Figure 5C).

The structural differences were evaluated for use as anode materials in lithium‐ion batteries with a loading mass of 0.7–0.8 mg cm^−2^. In the first cycle, galvanostatic charge/discharge curves were obtained in a voltage range of 0.005–1.5 V (Figure 5D). Exploiting Si as an anode active material poses challenges due to enormous volumetric expansion during electrochemical cycling, leading to structural instability.^[^ ^50^, ^51^ ^]^ The internal stress generated by the large volumetric changes can cause mechanical fracturing of the Si particles, rendering them ineffective as active materials.^[^ ^52^ ^]^ Moreover, the nanosized SiNP and MAS exhibit a much larger surface area, producing more unavoidable side reactions with the electrolyte and thus the formation of a further solid electrolyte interphase (SEI) layer on the interface.^[^ ^53^, ^54^ ^]^ Despite any pore generation in AAS and ZAS structures, the microspheres have relatively smaller surface area with fewer side reactions with the electrolyte, consistent with BET/BJH analysis results.^[^ ^55^ ^]^ SiNP and MAS showed reversible capacities of 2967.0 and 3082.4 mAh g^−1^ with initial Coulombic efficiencies of 89.1% and 89.7%, respectively. In contrast, with relatively smaller pore sizes, AAS and ZAS delivered high discharge capacities of 3140.3 and 3221.2 mAh g^−1^ with improved initial Coulombic efficiencies of 91.7% and 92.9%, validating higher reversibility for lithium‐ion. Interestingly, the SiNP and MAS electrodes retained 81.3% and 88.7% of their capacity over 50 cycles, and 40.1% and 63.5% over 100 cycles at 0.5C (1C = 2967.0 mA g^−1^ for SiNP and 3082.4 mA g^−1^ for MAS), respectively (Figure 5E). In the absence of hydrofluoric acid (HF) treatment, MAS allows unremoved impurities to remain and potentially provide structural stabilization against the volumetric changes, achieving better cycle stability than the SiNP electrode. However, the secondary structure of AAS and ZAS ensures compelling battery performances with capacity retention of 77.5% and 90.8% after 100 cycles at 0.5C (1C = 3140.3 mA g^−1^ for AAS and 3221.2 mA g^−1^ for ZAS), along with 61.3%, 71.0% after 150 cycles, respectively. Although AAS and ZAS possess similar hollow sphere shapes, variations in porosity play a crucial role in determining the electrochemical performance of the anode materials.^[^ ^56^, ^57^ ^]^ Moreover, the primary nanoparticulate structure of SiNP and MAS is relatively less stable than the secondary hollow and porous structure of AAS and ZAS, proving that internal voids and pores with moderate surface area act as a stress‐buffering space against volume expansion and thus effectively preserve the structural integrity of the electrodes during cycling.

## 3 Conclusion

The step‐by‐step reaction mechanism of molten salt‐modified metallothermic reduction, facilitated by AlCl_3_ salt, was unraveled from the atomic to the molecular level through detailed experimental investigations and rigorous DFT calculations. Under the unique chemistry of the molten AlCl_3_, the AlCl_3_ medium changes the thermodynamic energy level for the chemical reaction, even when deploying typically unfavorable metal reductants such as Zn. Within the molten AlCl_3_ matrix, all metals form metal‐AlCl_3_ complexes and then undergo internal Cl^*^ transfer, switching the role of all the metal reductants to Cl acceptors. The resultant AlCl^*^ disengages one oxygen atom on the SiO_2_ surface, releasing AlOCl. In the early stage of the reaction, metals with a high oxidation tendency can assist AlCl_3_ in direct oxygen dissociation, generating the same byproducts and the complex. Despite their common development into spherical Si shapes during the reaction, the accompanying metal mediates the stability of the remaining part of the complexes, rearrangement possibilities, and also the chemical composition of grown‐up Si, yielding structural dissimilarity in the morphology and porosity of the Si. To corroborate the distinctive characteristics, the electrochemical assessment was conducted utilizing AAS, MAS, and ZAS as anode materials. By exploiting metals tailored to the specific requirements in the molten AlCl_3_ salt medium, each produced Si offers excellent potential for application from secondary batteries to various fields including semiconductors, solar cells, and photo/photoelectric systems. Furthermore, the scalability of metallothermic reduction reaction could ensure the capability for practical production, satisfying the critical requirement of reducing manufacturing costs on a commercial scale.

## 4 Experimental Section

### Materials

Silicon dioxide (SiO_2_, 60–80 nm, Alfa‐Aesar), aluminum (Al, 1–3 µm, Avention), magnesium (Mg, 800 nm, Avention), zinc (Zn, 500 nm, US‐Nano), aluminum chloride (AlCl_3_, 98.5%, ACROS Organics), and hydrochloric acid (HCl, 35–37%, Samchun Chemical) were used without any further purification.

### Synthesis of AAS, MAS, and ZAS

For the preparation of aluminum/aluminum chloride‐reduced silicon (AAS), SiO_2_, Al, and AlCl_3_ were mixed in a mass ratio of 1 : 0.8 : 8 in an Ar‐filled glovebox. Similarly, for the production of magnesium/aluminum chloride‐reduced silicon (MAS) and zinc/aluminum chloride‐reduced silicon (ZAS), SiO_2_, Mg or Zn, and AlCl_3_ were mixed in a mass ratio of 1 : 3 : 30 or 1 : 5 : 20, respectively. The mixtures were then transferred to a custom‐made reactor where the reduction reaction was carried out at 250 °C for a duration of 20 h. The as‐reduced sample was sequentially rinsed with deionized water and 1 m HCl acidic solution after natural cooling to room temperature. Finally, the samples were filtered using deionized water and dried in a vacuum at 70 °C overnight.

### Physical Characterization

The morphology, size distribution, and elemental characterization were analyzed through field‐emission scanning electron microscopy (FE‐SEM, S‐4800, Hitachi). The crystal structure was studied using X‐ray diffraction (XRD) analysis (SmartLab, Rigaku). Time‐of‐flight secondary ion mass spectrometry (TOF‐SIMS, TOF‐SIMS 5, ION TOF) and X‐ray photoelectron spectroscopy (XPS, K‐alpha, ThermoFisher) were adopted to track the creation and consumption process of metal/AlCl_3_ complexes, as well as the corresponding byproducts, as the reaction proceeded. TOF‐SIMS analysis was conducted under the conditions of using Bi^+^ (25 keV, 1.1 pA) as a primary beam in a vacuum environment (<5.0 × 10^−10^ Torr). A home‐built confocal micro‐Raman system was employed to identify changes of the Al─Cl and Zn─Cl bondings inside the Zn‐AlCl_3_ complex with an excitation wavelength of 514 nm. Raman signals were collected in back‐scattering geometry, focused through the objective lens, and dispersed using a spectrometer (iHR550, HORIBA Scientific). Furthermore, another confocal Raman spectrometer (alpha 300R, Witec) was utilized to verify the formation of Mg‐Si bonds in MAS with an excitation wavelength of 488 nm. Auto Physisorption Analyzer (ASAP2020 Analysis, Micromeritics Instruments) was employed for BET and BJH analyses to examine surface area, pore size, and pore volume, in order to differentiate structural properties.

### Computational Modeling & Simulation Details

The molten AlCl_3_ salt‐modified Si‐reduction mechanism over a silicon oxide surface using Dmol^3^ program was investigated.^[^ ^58^, ^59^ ^]^ All reduction mechanisms were predicted to involve a two‐step reaction: the formation of metal‐AlCl_3_ complexes where molten AlCl_3_ clusters detached atomic Mg or Zn metal from each metallic surface, and the reduction of silicon oxide by the adsorbed metal‐AlCl_3_ complex. The most stable (001) surfaces of Zn and Mg were adopted when exploring the metal complexes in the calculation. The silicon oxide surface was represented by monolayer kaolinite $\left(00\bar{1}\right)$ because the reduction reaction occurs with decomposed fragments from silicon precursors.^[^ ^17^ ^]^ Despite a layered silicon oxide structure consisting of a corner‐sharing SiO_4_ sheet linked to an edge‐sharing AlO_6_ sheet, only reactions on the SiO_4_ sheet surface (Figure S19, Supporting Information) were considered for mechanism calculation to readily observe the formation of a new Si‐Si bond. For the DFT calculation, the Perdew‐Burke‐Ernzerhof (PBE) exchange‐correlation functional and DNP 4.4 basis set with the all‐electron relativistic core treatment were utilized.^[^ ^60^ ^]^ The convergence criteria for energy, force, and displacement were set to 1 × 10^−5^ Ha, 0.002 Ha/Å, and 0.005 Å, respectively. The Tkatchenko‐Scheffler scheme was used to include the dispersion correction of the van der Waals effect.^[^ ^61^ ^]^ The Brillouin zone was sampled by a Monkhorst‐Pack for a single k‐point (G‐point) in all model systems. To calculate transition states in the mechanism of Si reduction, complete single linear synchronous transit (LST) and quadratic synchronous transit (QST) methods were employed, setting the convergence criteria for the root mean square (RMS) force to 0.003 Ha/Å.^[^ ^62^, ^63^ ^]^

### Electrochemical Characterization

All anodes were prepared on copper (Cu) foil through a slurry‐casting method. The slurry was formulated by mixing active materials, binder, and Super P in a mass ratio of 60:20:20, and poly(acrylic acid) (PAA)/carboxymethyl cellulose (CMC) (weight ratio of 1:1) was used as a binder. This homogenous slurry was cast onto Cu foil with a loading mass of 0.7–0.8 mg cm^−2^ and then dried in a vacuum oven at 150 °C for 2 h. The coin‐type cells (CR2032, Welcos), which were assembled in an Ar‐filled glove box, were utilized to evaluate the electrochemical performance of the fabricated anodes. The coin‐type cells incorporated polypropylene membrane (Celgard 2400) as a separator, 1.3 m lithium hexafluorophosphate (LiPF_6_) in ethylene carbonate (EC)/diethyl carbonate (DEC) (3 : 7 v/v) including 10 wt.% fluoroethylene carbonate (FEC) additive as an electrolyte, and lithium metal as counter/reference electrode. All the electrochemical characterizations were carried out using a galvanostatic battery cycler (WBCS3000, Wonatech) with a potential window of 0.005–1.5 V for a formation cycle and 0.01–1.2 V for subsequent cycles.

## Conflict of Interest

The authors declare no conflict of interest.

## Supporting information



Supporting Information

## Data Availability

The data that support the findings of this study are available from the corresponding author upon reasonable request.
