# Supplementary material for: Metal‐Mediated Chlorine Transfer for Molten Salt‐Driven Thermodynamic Change on Silicon Production
Source: Adv Sci (Weinh). 2024 Dec 3;12(4):2412239. doi: 10.1002/advs.202412239 (PMC11775547; doi:10.1002/advs.202412239)
Supplement: Supplementary file 1 — Supporting Information [file ADVS-12-2412239-s001.docx]

Supporting Information

**Metal-Mediated Chlorine Transfer for Molten Salt-Driven Thermodynamic Change on Silicon Production**

*Minjun Je, Jin Chul Kim, Jiyeon Kim, Sungho Kim, Sunmin Ryu, Jaegeon Ryu,* Sang Kyu Kwak,* Soojin Park**


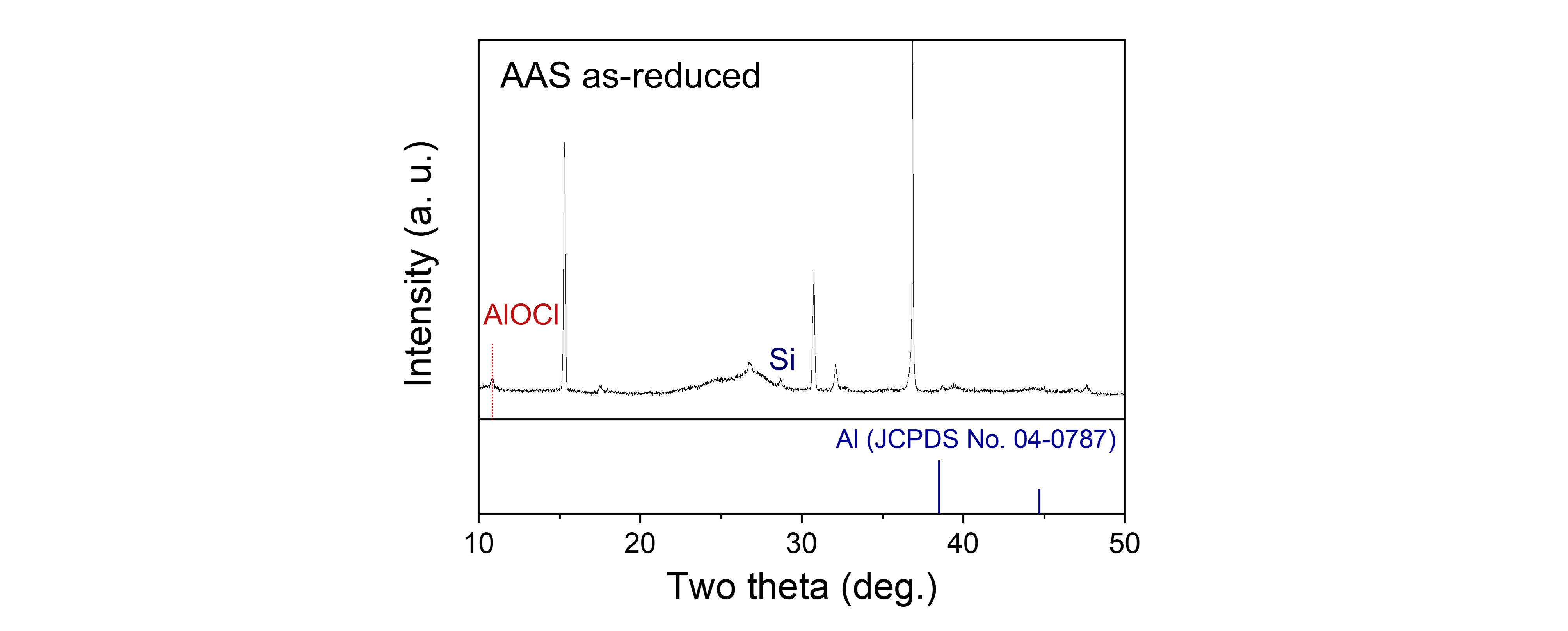


**Figure S1.** XRD pattern of AAS as-reduced sample.


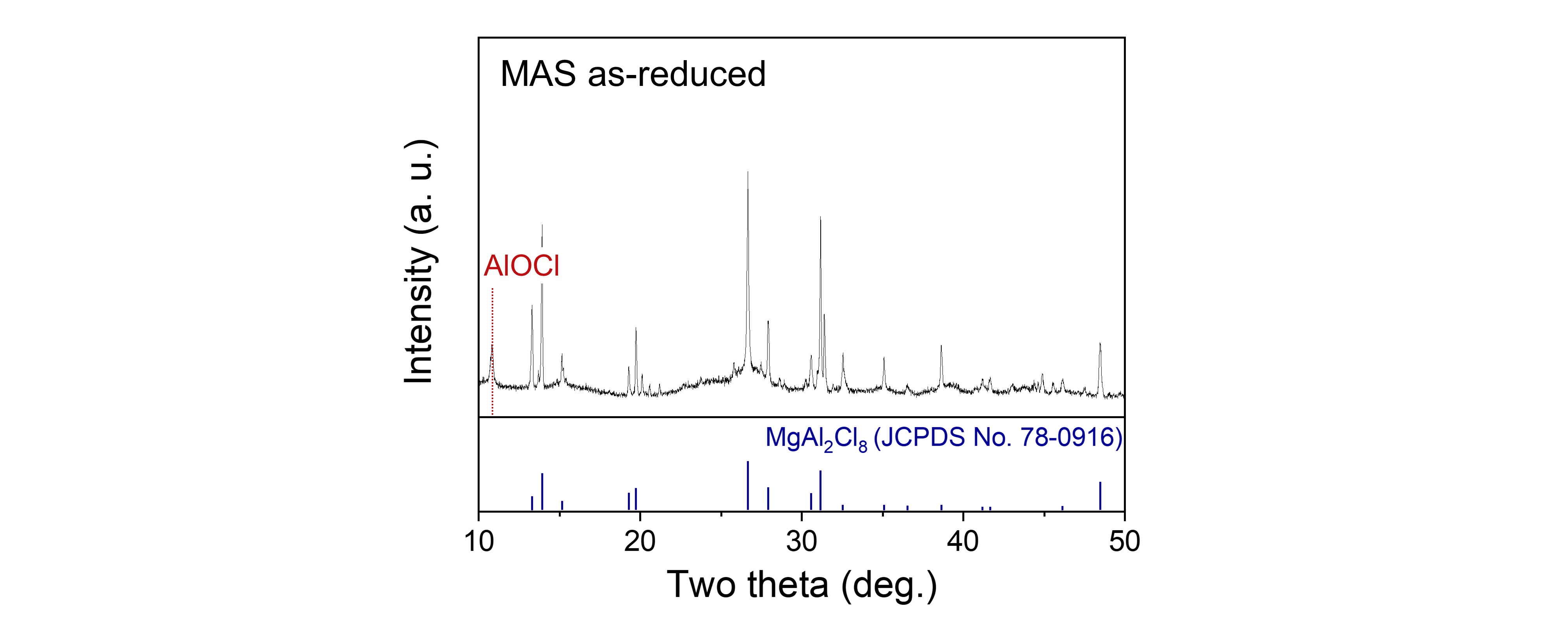


**Figure S2.** XRD patterns of MAS as-reduced sample.


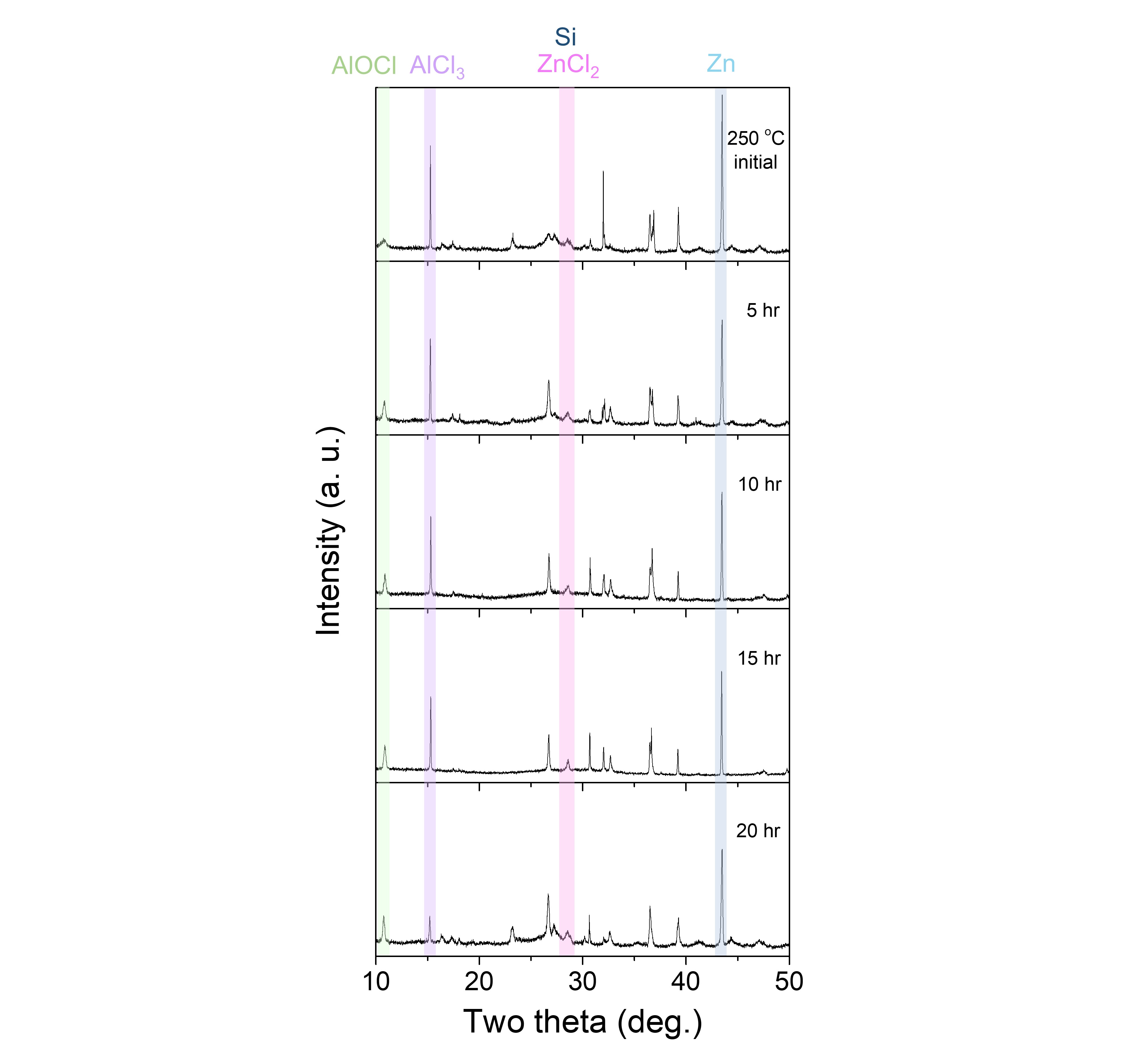


**Figure S3.** XRD patterns of samples participated in AlCl_3_ molten salt-modified zincothermic reduction reaction for 250 ℃ initial, 5 hours, 10 hours, 15 hours, and 20 hours.


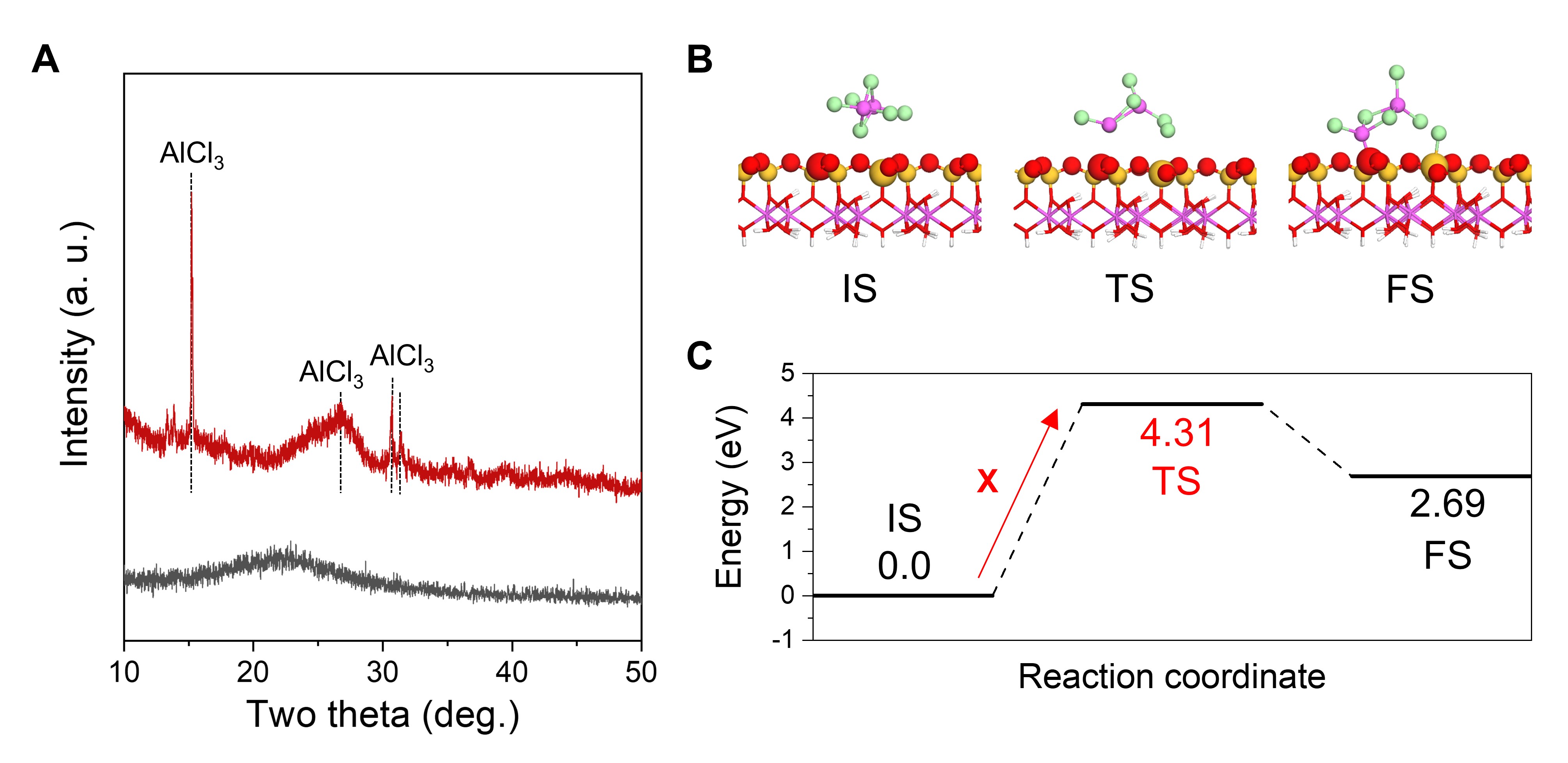


**Figure S4.** (A) XRD patterns of bare SiO_2_ (black) and SiO_2_ + AlCl_3_ as-reacted sample (red) and corresponding (B) configurations and (C) theoretical calculation results of each state for sole AlCl_3_ molten salt (without any metals) to occur reduction reaction of SiO_2_ at the same condition. For a clear view, the top layer is presented in different styles, i.e. ball-and-stick. For the color scheme of atoms, Al and Cl atoms are colored in pink and light green, and Si, O, and H atoms are colored in yellow, red, and white, respectively. The numbers represent the relative energies of each state based on that of the IS.


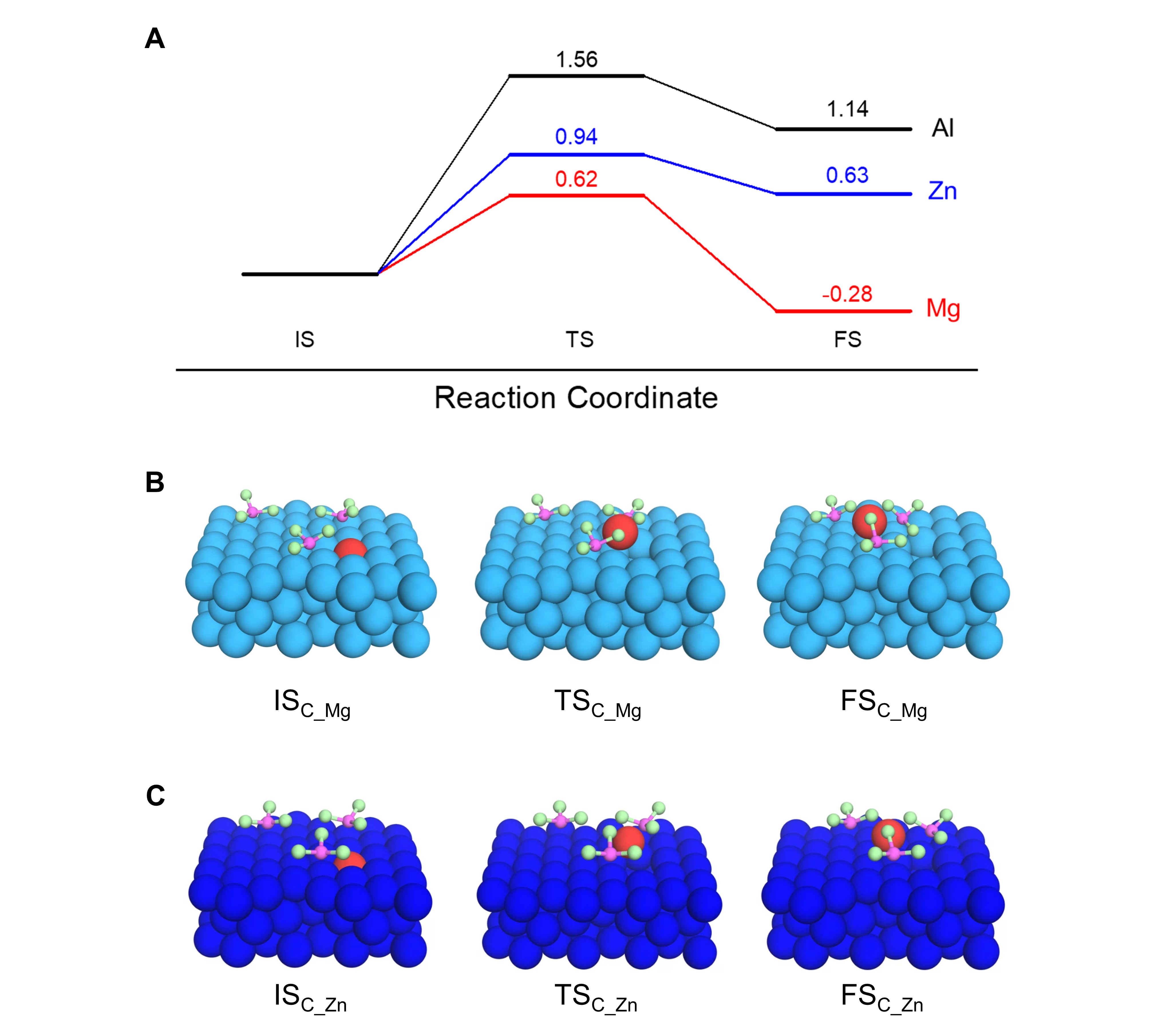


**Figure S5.** (A) Reaction coordinates and the configurations of (B) Mg and (C) Zn complexes formation reaction. For the color scheme of atoms, Al and Cl atoms are colored in pink and light green, and Mg, Zn, and metal center atoms are colored in sky blue, blue, and red, respectively. The numbers represent the relative energies of each state based on that of the IS.


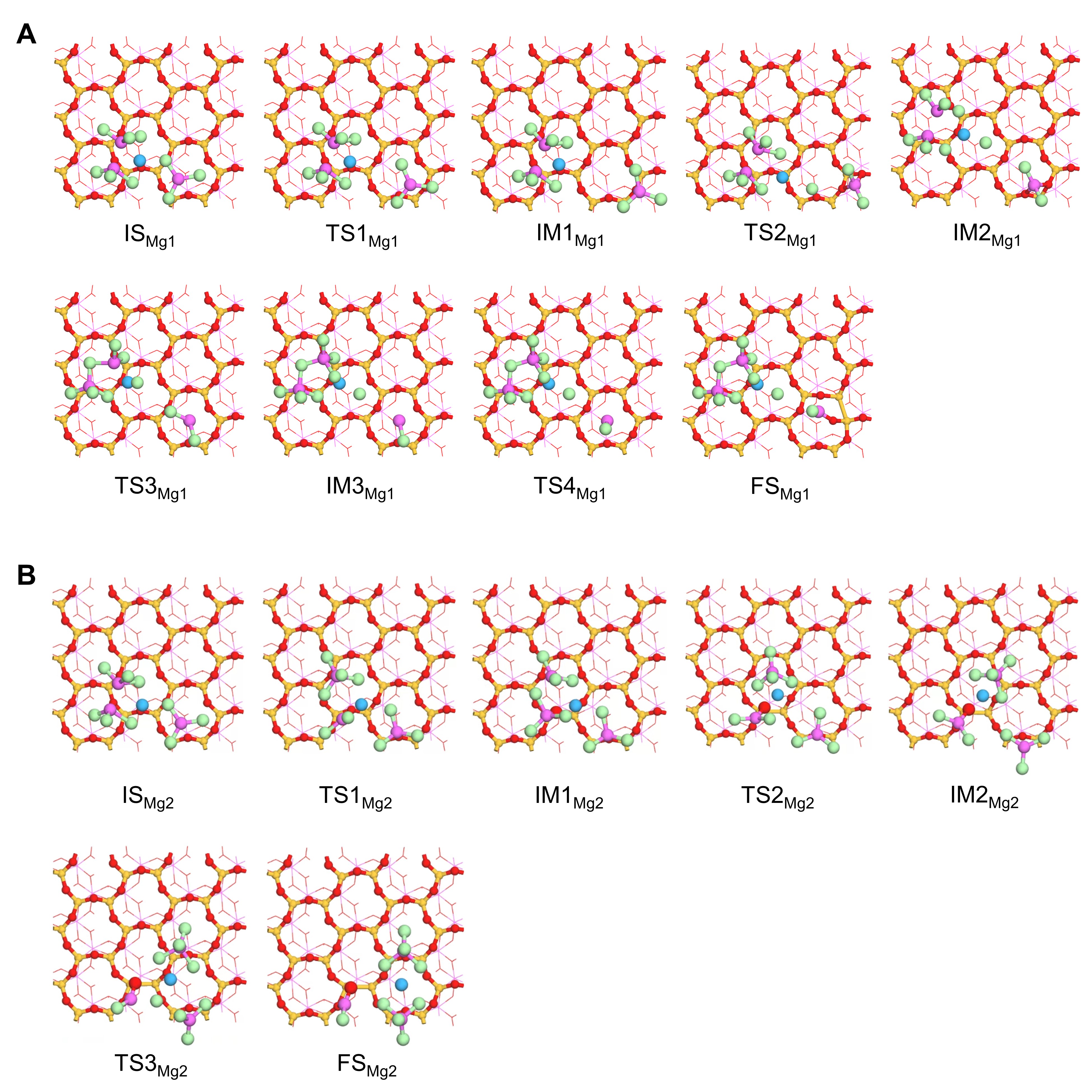


**Figure S6**. The reaction configurations of (A) *route 1* and (B) *route 2* of AlCl_3_ molten salt-modified magnesiothermic reduction. For the color scheme of atoms, Mg, Al, and Cl atoms are colored in sky blue, pink, and light green, and Si, O, and H atoms in the silicon oxide are colored in yellow, red, and white, respectively.


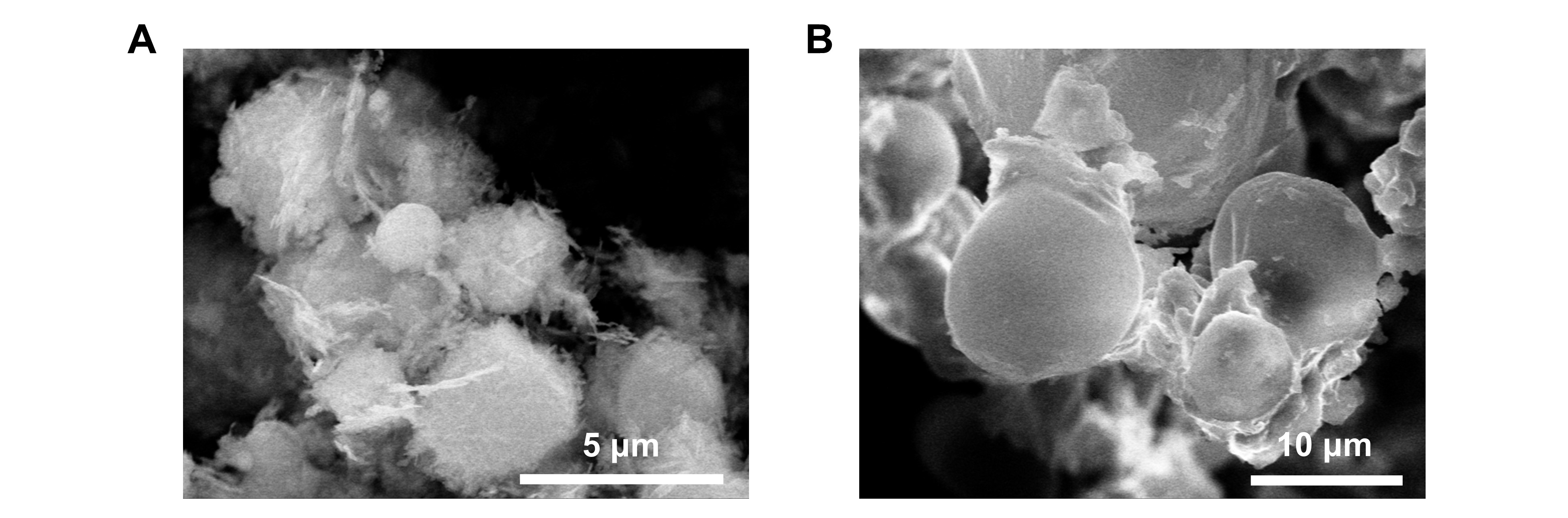


**Figure S7**. SEM images of as-reduced (A) AAS and (B) ZAS particles without any treatment.


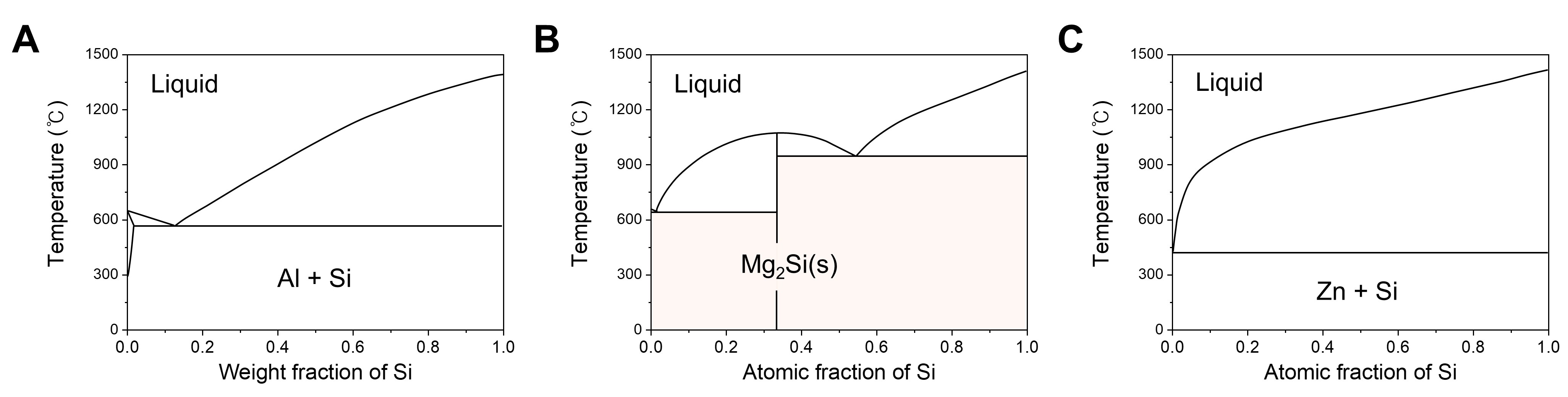


**Figure S8.** Calculated phase diagram of (A) Al-Si, (B) Mg-Si, and (C) Zn-Si.


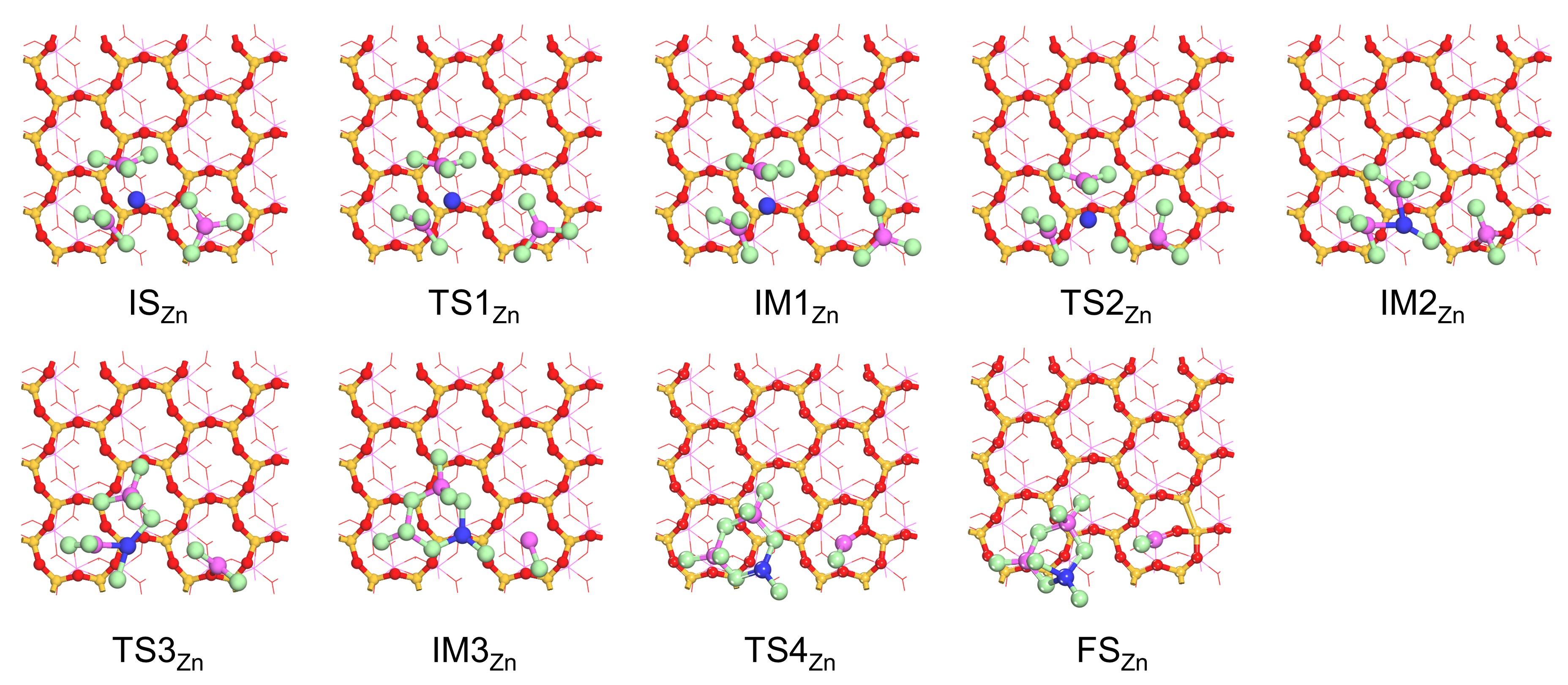


**Figure S9**. The reaction configurations of IS_Zn_, TS_Zn_, IM_Zn_, and FS_Zn_ of AlCl_3_ molten salt-modified zincothermic reduction. For the color scheme of atoms, Zn, Al, and Cl atoms are colored in blue, pink, and light green, and Si, O, and H atoms in the silicon oxide are colored in yellow, red, and white, respectively.


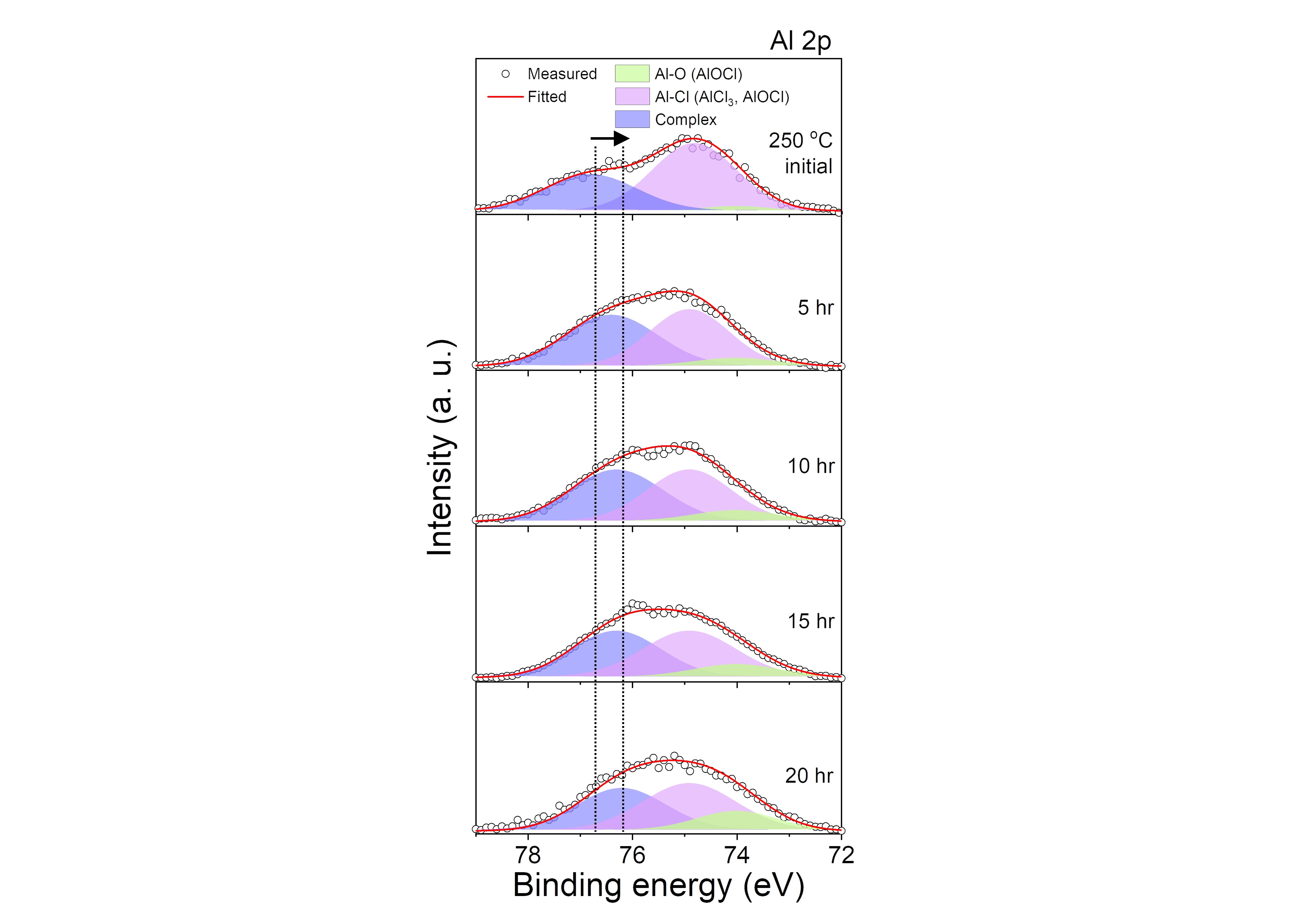


**Figure S10.** Al 2p XPS spectra of samples participated in AlCl_3_ molten salt-modified zincothermic reduction reaction for 250 ℃ initial, 5 hours, 10 hours, 15 hours, and 20 hours.


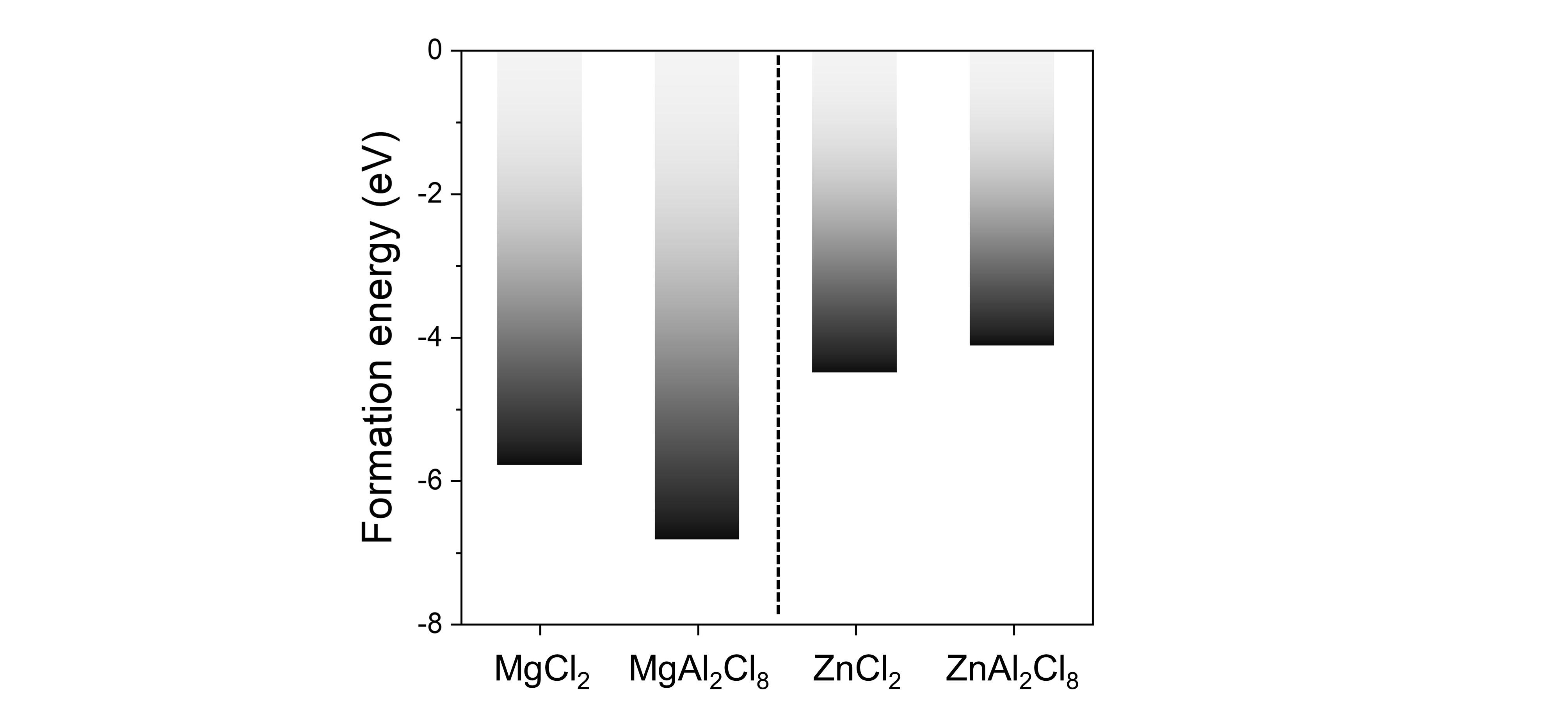


**Figure S11.** Formation energy of ZnCl_2_, MgCl_2_, and each metal-AlCl_3_ complex.


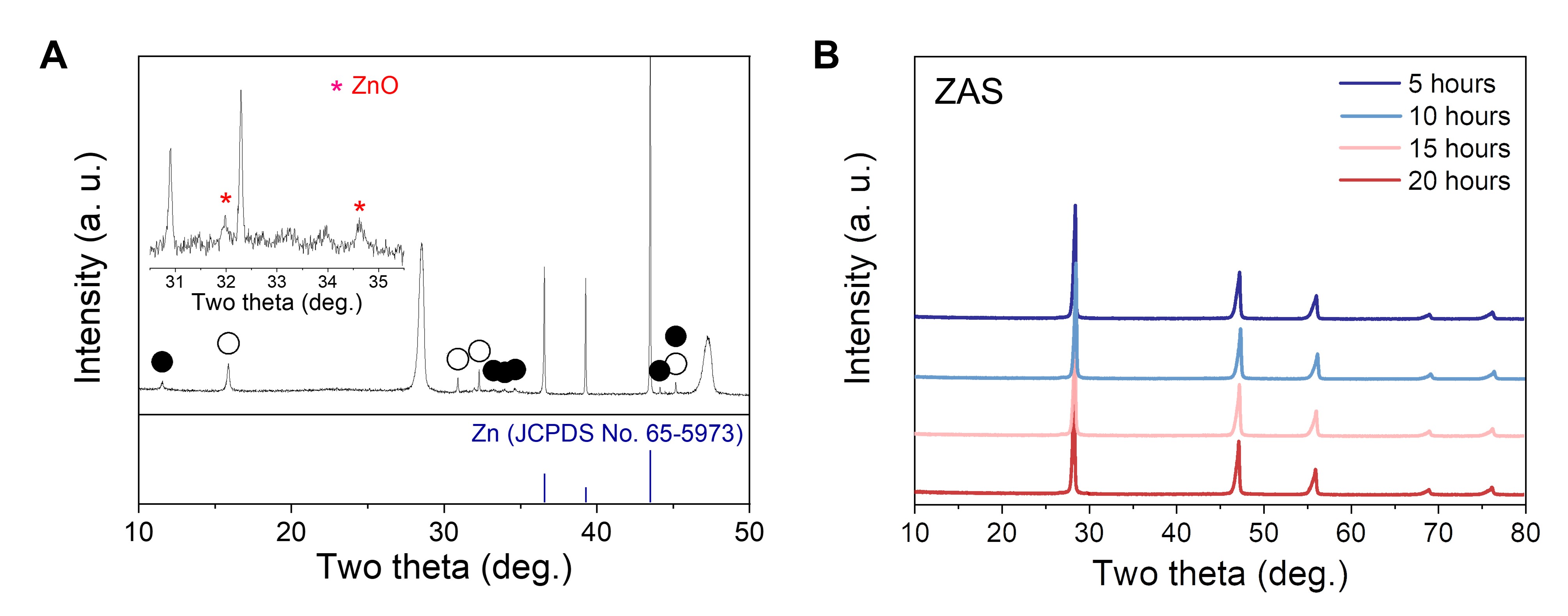


**Figure S12.** XRD patterns of water-treated (INT-2) samples after AlCl_3_ molten salt-modified zincothermic reduction reaction for (A) 250 ℃ initial, and (B) 5 hours, 10 hours, 15 hours, and 20 hours (●: Zn_5_(OH)_8_Cl_2_·(H_2_O), ○: β-Zn(OH)Cl).


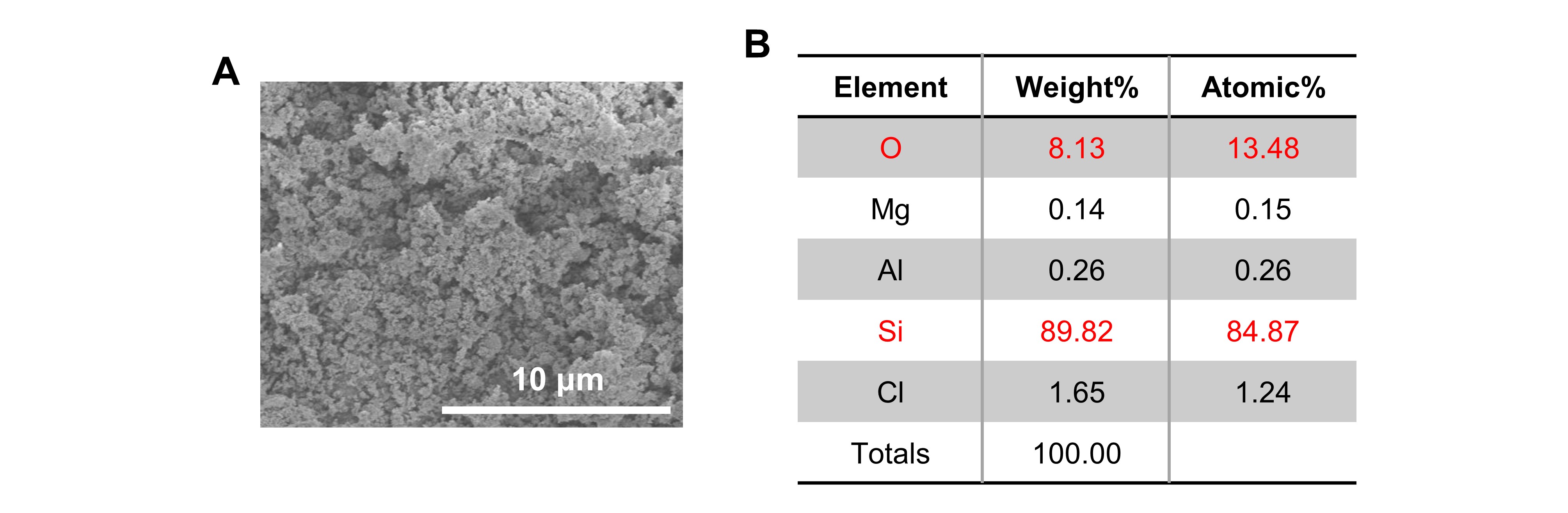


**Figure S13.** (A) Low-magnified SEM image and (B) elemental characterization of HCl-treated MAS sample in AlCl_3_ molten salt-modified magnesiothermic reduction reaction for 20 hours.


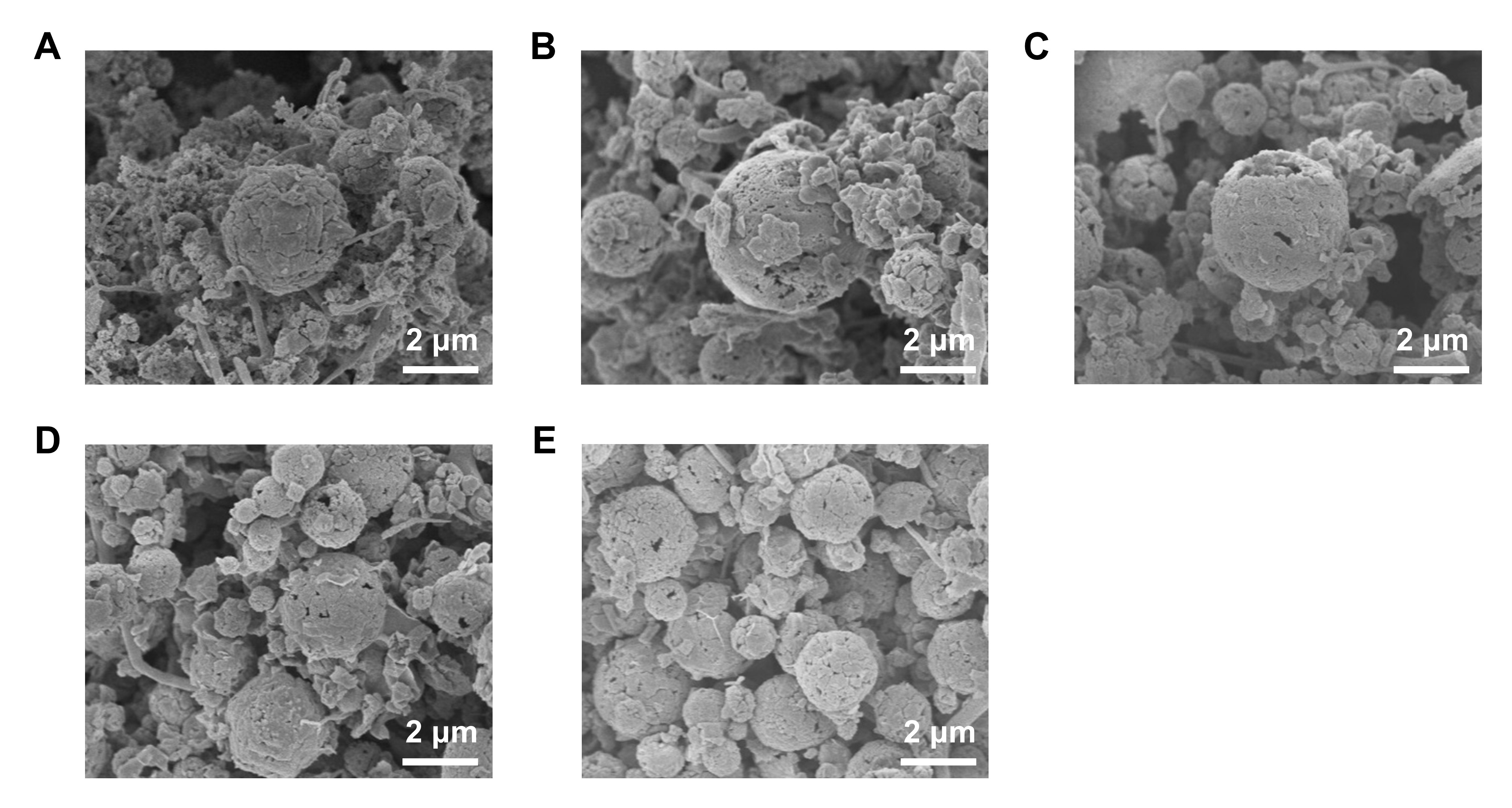


**Figure S14.** SEM images of water-treated (INT-2) ZAS samples after AlCl_3_ molten salt-modified zincothermic reduction reaction for (A) 250 ℃ initial, (B) 5 hours, (C) 10 hours, (D) 15 hours, and (E) 20 hours.


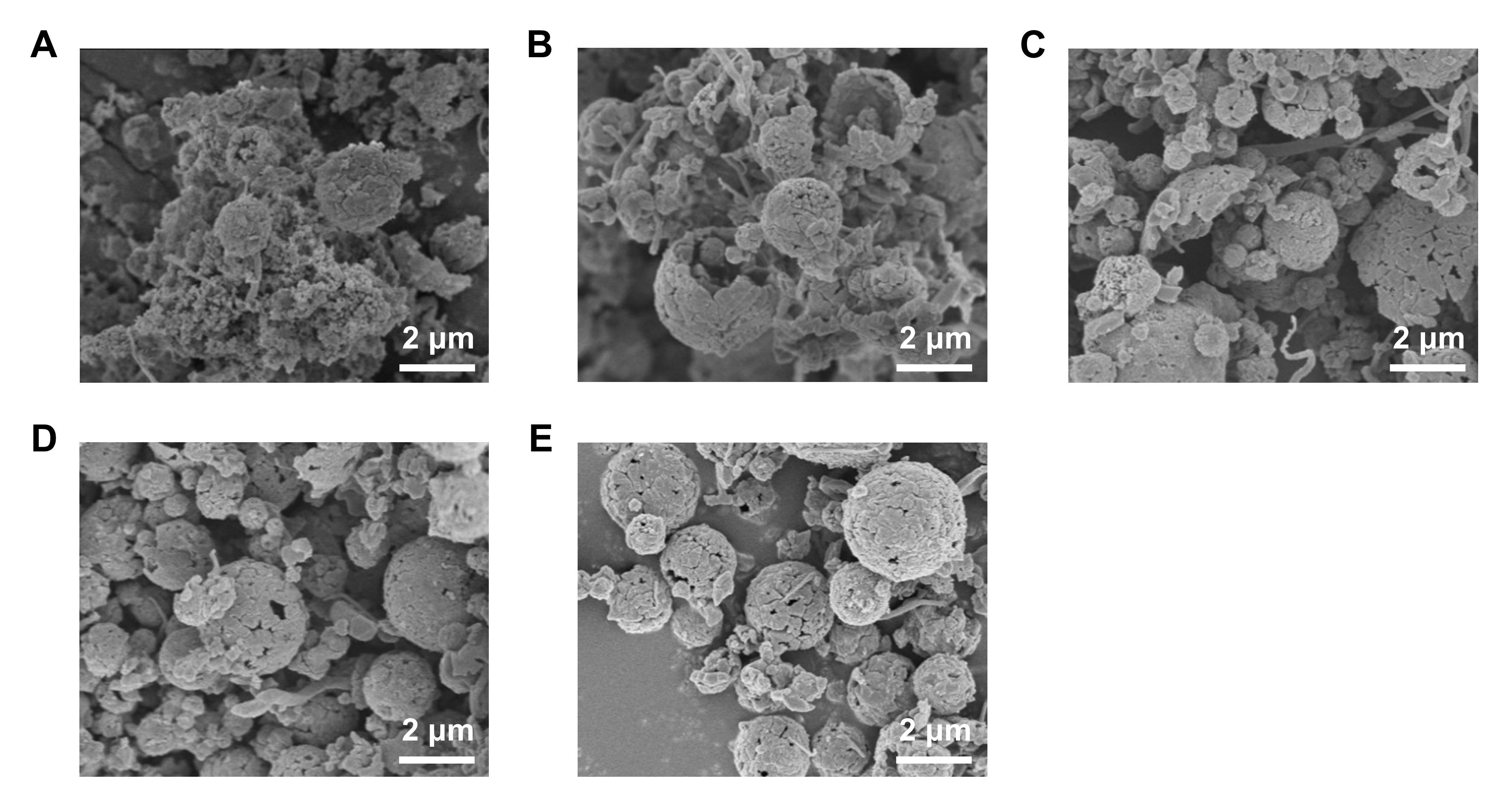


**Figure S15.** SEM images of HCl-treated ZAS samples after AlCl_3_ molten salt-modified zincothermic reduction reaction for (A) 250 ℃ initial, (B) 5 hours, (C) 10 hours, (D) 15 hours, and (E) 20 hours.


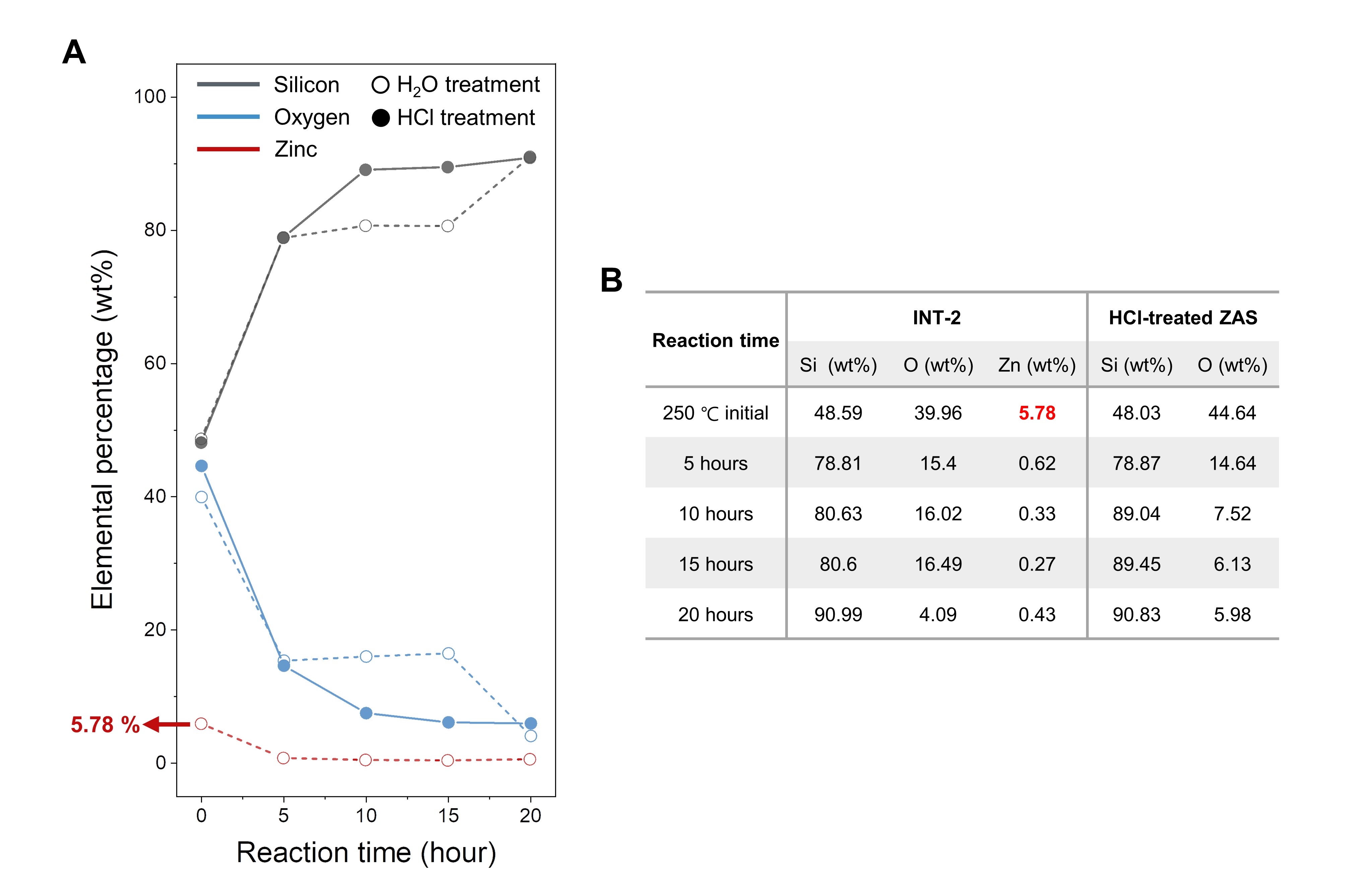


**Figure S16.** Elemental characterization of water-treated (INT-2) or HCl-treated ZAS samples in AlCl_3_ molten salt-modified zincothermic reduction reaction for 250 ℃ initial, 5 hours, 10 hours, 15 hours, and 20 hours.


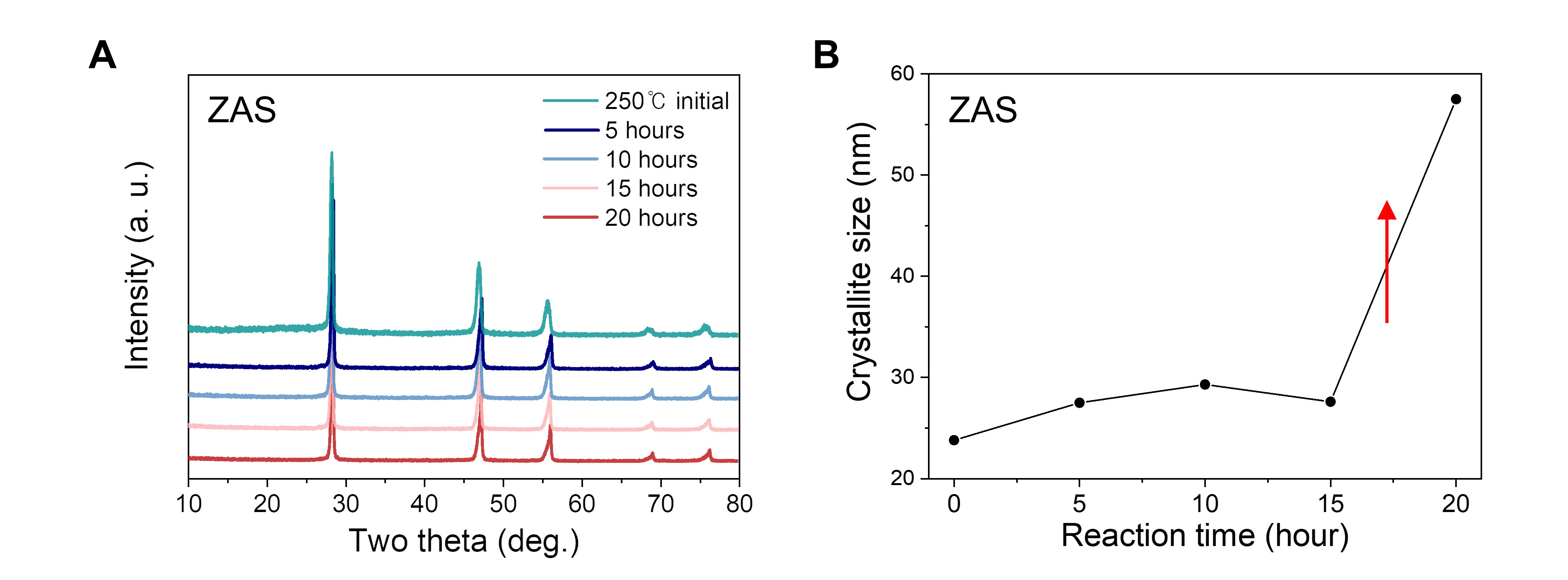


**Figure S17.** (A) XRD patterns and (B) corresponding crystallite size comparison of HCl-treated ZAS samples after AlCl_3_ molten salt-modified zincothermic reduction reaction for 250 ℃ initial, 5 hours, 10 hours, 15 hours, and 20 hours.


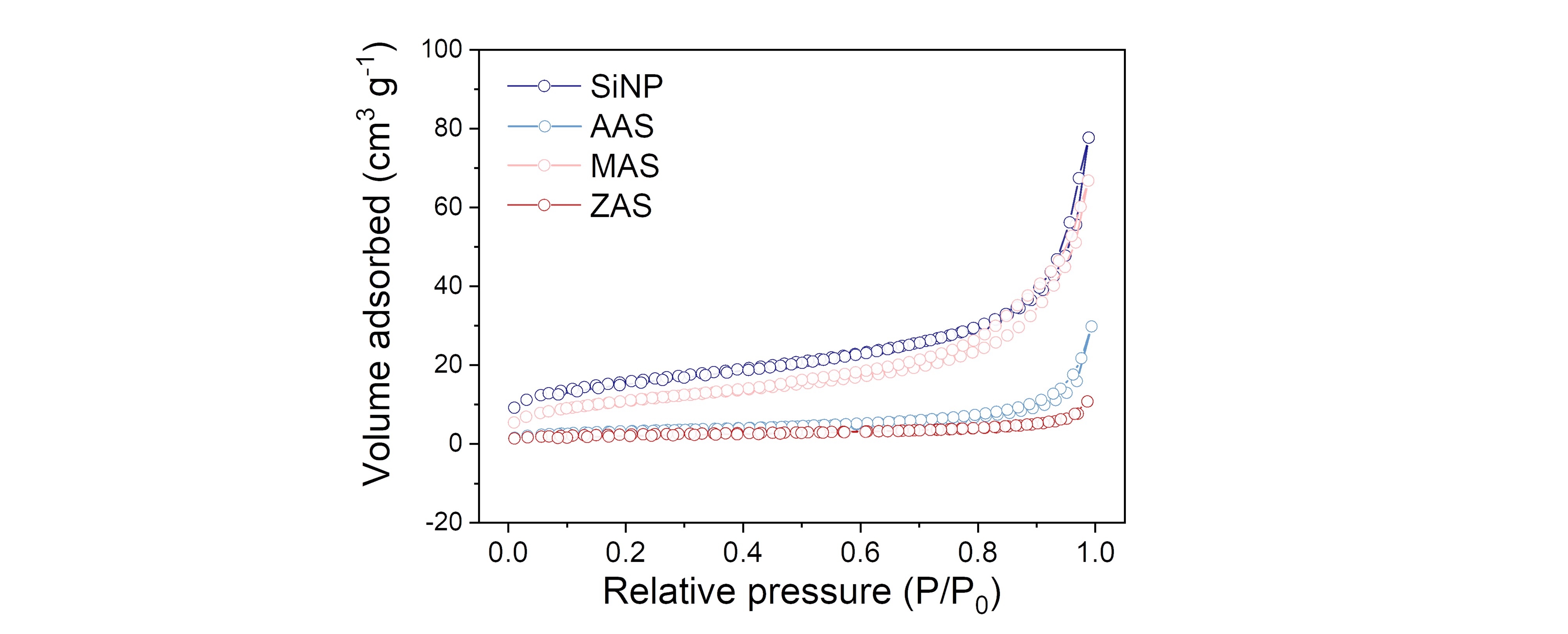


**Figure S18.** Nitrogen adsorption/desorption analysis of SiNP, AAS, MAS, and ZAS.


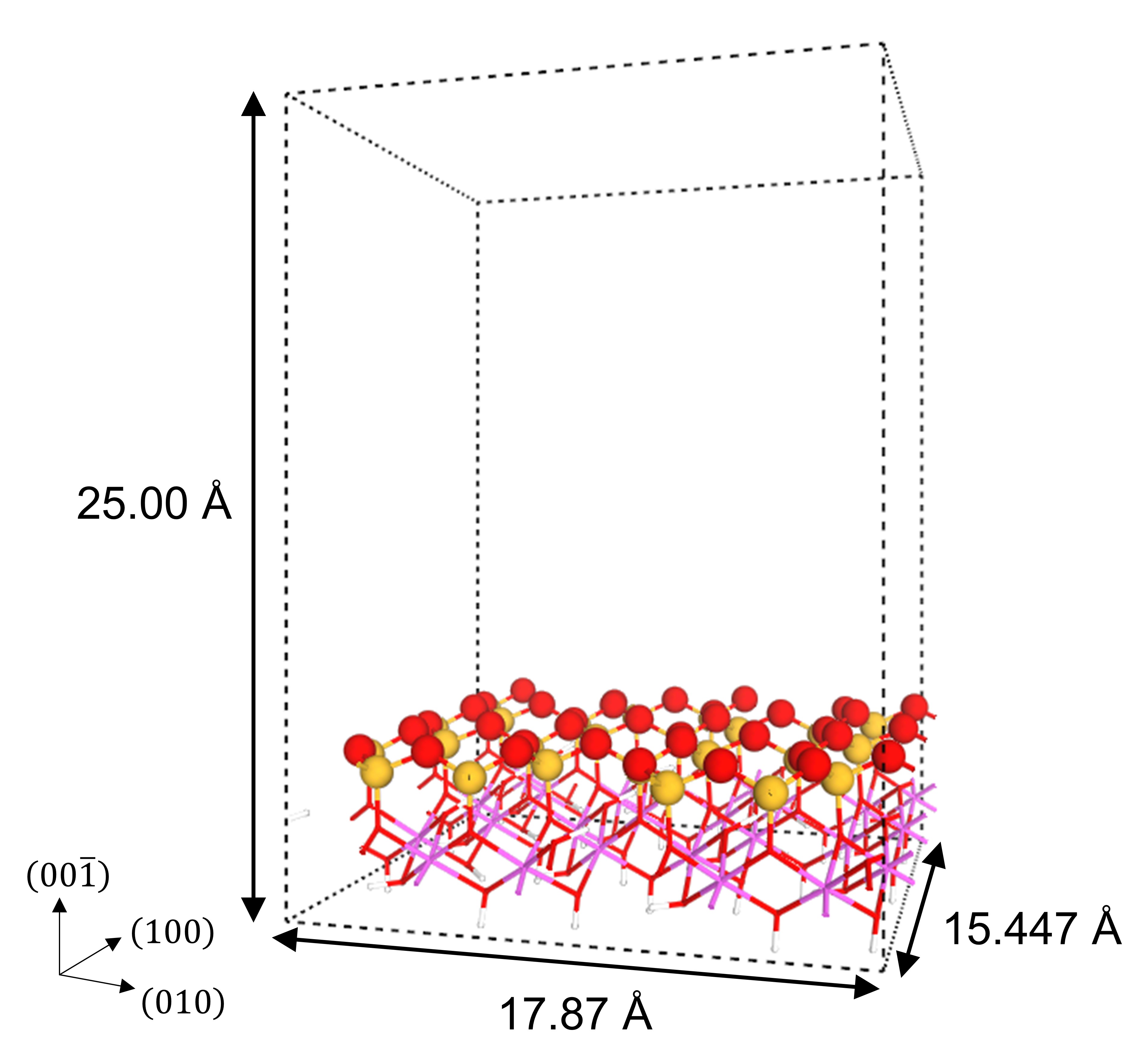


**Figure S19.** Calculation model of the representative silicon oxide material, kaolinite (001̅). For a clear view, the top layer is presented with different styles, i.e. ball-and-stick. For the color scheme of atoms, Al and Si atoms are colored in pink and yellow, and O and H atoms are colored in red and white, respectively. Note that the bottom layer was fixed to represent the bulk phase, and we optimized the atoms at the outmost surface.
